# B,N‐Doped Activated Carbon‐Based Electrodes from Potato Peels for Energy Storage Applications

**DOI:** 10.1002/open.202400527

**Published:** 2025-02-19

**Authors:** Jan Willem Straten, Muhammad‐Jamal Alhnidi, Ghassan Alchoumari, Krishna Sangam, Andrea Kruse

**Affiliations:** ^1^ University of Hohenheim Institute of Agricultural Engineering Department of Conversion Technologies of Biobased Resources Garbenstr. 9 70599 Stuttgart Germany

## Abstract

Potato peels (PPs) as waste biomass were selected as the biobased carbon source for this study, using urea as N precursor and boron trioxide as B precursor for the “in situ doping” via hydrothermal carbonization (HTC). During HTC, the feedstocks decompose over a wide range of complex chemical degradation mechanisms that finally form single B‐ and N‐ as well as B,N‐co‐doped hydrochars (HCs). Upon chemical ZnCl_2_ activation, the single B‐doped activated carbon (AC) possessed a maximum B content of 0.2 wt%, whereas co‐doped B,N‐AC had the highest N content of 5.7 wt% with a B content of 0.1 wt%. The influence of single and B,N‐co‐doping on the physical‐chemical material properties of the AC electrodes was analyzed and compared, in combination with its effect on the electrochemical performance for energy storage application. Compared to pristine AC derived from PPs, the B‐doped and B,N‐co‐doped AC depicted increased electrical conductivity (EC) values of 50.3 S ⋅ m^−1^ and 34.0 S ⋅ m^−1^, respectively. In addition, the B,N‐co‐doped AC unveiled the highest average specific capacitances of 51.7 F ⋅ g^−1^ at 100 mV ⋅ s^−1^ and of 71.9 F ⋅ g^−1^ at 5 mV ⋅ s^−1^ outperforming the specific capacitance values of the reference material AC from peat.

## Graphical abstract

B,N‐co‐doped activated carbon electrode (AC‐PPs‐B‐N) was synthesized from potato peels and dopants of N and B precursors by means of hydrothermal carbonization and subsequent chemical activation. This turned out to be an appealing candidate for energy storage applications, whose electrochemical performance even exceeds that of the benchmark (AC‐Peat).

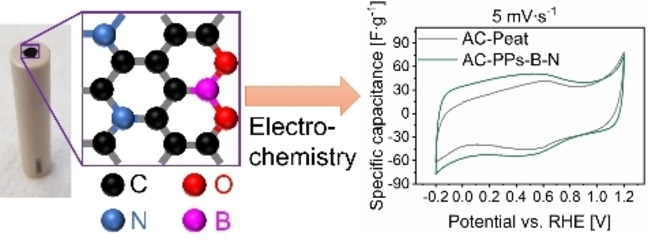

## 1 Introduction

The fast development in (portable) electronic systems, such as electrochemical energy storage (e. g. batteries and supercapacitors) and energy conversion devices (e. g. fuel cells), is accompanied by a steady increase in energy demand in everyday life. Besides batteries, supercapacitors are currently the most crucial energy storage devices due to their unique benefits. These include high power density, low internal resistance, wide temperature range with stable operation, good stability (as they are adaptable to harsh environmental conditions), high reliability, high round‐trip efficiencies, quick charge/discharge rates, and a long lifetime (cycle life), rendering them well‐suited for instant power supply and recharging.[^1^, ^2^, ^3^] A decisive factor is the electrode. The electrode material should be characterized by a pronounced graphitization degree, specific surface area (SSA), or surface properties that meet high electrochemical performance.^[3]^ Furthermore, the incorporation of heteroatoms into the carbon skeleton, such as nitrogen (N) and boron (B), provide promising electroactive sites provoking desirable electrical features, increased overall charge storage, and overall electrochemical performance.[^2^, ^3^, ^4^, ^5^, ^6^, ^7^] N as an n‐type dopant is an electron donor. N alters the carbon surface chemical reactivity by increasing wettability, which is beneficial for electrical conductivity (EC) and capacitance behavior.^[8]^ B as a p‐type dopant is an electron acceptor. B atoms entail an increase in the degree of graphitization of non‐graphitizable carbons elevating significantly the conductivity.[^5^, ^6^, ^8^] Furthermore, B also enhances the capacitance performance.^[9]^ The combination of both heteroatoms provides desirable synergetic effects. Co‐doping or dual‐doping leads to a unique electron distribution and structural distortions that may improve the electrochemical performance.[^3^, ^8^] The B,N‐co‐doped carbon electrodes raise the defect sites in the carbon scaffold and not only augment but also bear structurally complex active sites. These active sites impart the carbon structure to more advantageous chemical properties compared to single‐doping, such as enhanced reactivity, improved catalytic/chemical/electrochemical activity, better wettability for increased electrode‐electrolyte interaction, superior electronic properties, enhanced EC, high energy density, low ohmic and charge transfer resistance as well as outstanding cyclic stability (longer lifetime).[^2^, ^5^, ^6^, ^7^] In connection with the growing demand for securing affordable, clean, and efficient energy sources, carbon‐based electrode materials from renewable resources have proven to be viable alternatives to fossil raw materials.[^3^, ^10^, ^11^] The hydrothermal synthesis, also referred to as hydrothermal carbonization (HTC), is a well‐established exothermic thermochemical conversion process operating under subcritical water conditions utilized to transform (waste) biomass into a valuable, carbon‐rich solid material, commonly termed hydrochar (HC).[^8^, ^12^] HTC can tune the morphology, chemical composition, surface functionality, and structure of the HC.^[13]^ The HTC as an ideal sustainable synthesis method offers an optimal environment for heteroatom “in situ doping”.[^8^, ^14^]. A myriad of tailor‐made biomass‐derived B‐, N‐, S‐, P‐ or O‐single‐doped and co‐doped carbon electrodes for supercapacitor application have been studied. These include willow catkin,^[15]^ Macadamia nutshell,^[16]^ bean shell,^[8]^ or egg yolk.^[17]^

The main focus of this work is on potato peels (PPs), which are an abundant agricultural waste product. PPs are a natural self‐doped N‐ and O‐enriched waste biomass[^18^, ^19^, ^20^] that represent an appealing candidate for the synthesis of high‐performance, sustainable carbon‐based electrode materials. Thus, converting PPs into carbon‐based electrode materials for energy storage would be a vital contribution to a high added‐value application. Potato is the fourth most important cultivated plant after rice, wheat, and maize. It is decisive in human consumption worldwide.^[21]^ For instance, in 2019, potato production accounted for >370 million tons.^[22]^ About 6–10 % of potato production is generated as inedible PP waste due to peeling losses.[^21^, ^23^] PP waste is the main by‐product in potato processing and is rich in carbonaceous components (polysaccharides such as starch, proteins, and lipids) and nutrient content (K or Ca minerals), which makes it an attractive precursor for producing carbon materials.[^21^, ^23^] So far, PPs have not yet been utilized efficiently, which means that it is rather disposed of in landfills or processed into low added‐value fodder or fertilizer.^[21]^ Nevertheless, the PPs can also be valorized to generate biogas or alcohols (e. g. methanol and ethanol).^[24]^

**Figure 1:**
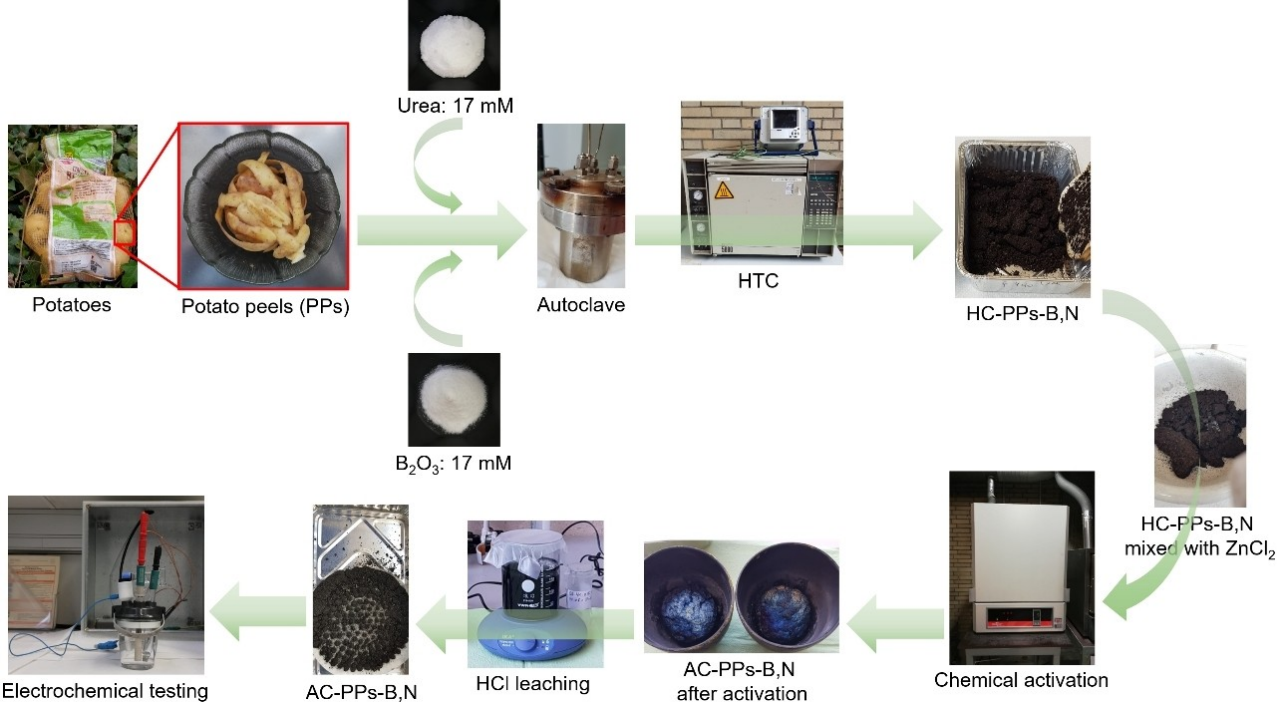
Schematic illustration outlining the synthetic pathway of B,N‐co‐doped AC electrodes derived from PPs.

Herein, the conversion of PPs into B‐, and N‐single as well as co‐doped carbon electrodes by means of HTC and chemical activation for energy storage applications has been investigated. HTC is coupled with the “in situ doping” method, in which the doping agents B_2_O_3_ as the B‐dopant and urea as the N‐dopant are utilized. Urea and B_2_O_3_ are characteristic dopants applied to enhance supercapacitive properties.[^25^, ^26^, ^27^] For the chemical activation of HCs, ZnCl_2_ has been applied as an activating agent. It is reported how the combination of self‐doped biomass with the “in situ doping” method and the subsequent chemical activation with ZnCl_2_ affects the physical‐chemical material properties of the AC electrodes for energy storage application. To the best of our knowledge, there is no study that deals with both single‐ and co‐doping of PPs. This work aims to shed light on whether the B‐, and N‐single as well as co‐doping of PP waste with the dopants B_2_O_3_ and urea has an effect on the physical‐chemical material properties and whether B,N‐co‐doping ultimately influences the electrochemical performance. The schematic procedure of the entire synthesis route of the heteroatoms‐doped porous AC from PP waste is shown in Figure 1.

## 2 Results and Discussion

### 2.1 Characterization of Hydrochars (HCs)

#### 2.1.1 Chemical Composition

**Figure 2:**
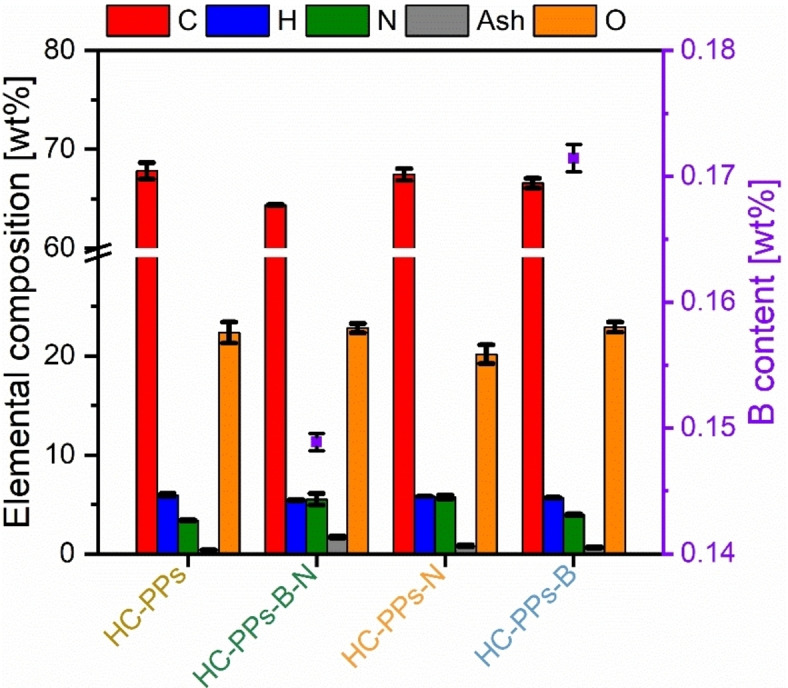
Elemental composition and ash content (columns) and B content (squares) after HTC of PPs with the corresponding B precursor B_2_O_3_ and the N precursor urea. The values of the S content were not taken into account in the graphical plot, as the values are <0.1 %.

To examine whether the “in situ doping” method with the corresponding B and N precursors (B_2_O_3_ and urea) was successful, the elemental composition of all HCs was characterized, as summarized in Figure 2 (Table S1 in the Supporting Information compile the absolute values).

The average N content of the pristine HC from potato peels (HC‐PPs) is 3.4 wt%. When urea is added, the N content rises. For the HC with a mixture of the dopants B_2_O_3_ and urea (HC‐PPs‐B‐N), the proportion increases to 5.5 wt%. The highest N content of 5.7 wt% was achieved for the single‐doped HC with urea (HC‐PPs‐N). When the B precursor B_2_O_3_ is added, there is no appreciable rise of the B content. For the two samples HC‐PPs‐B‐N and HC‐PPs‐B, the B percentage is at a similarly low level of 0.15 wt% and 0.17 wt% respectively. The C content is similarly high in all HCs varying between 64 wt% and 68 wt%. The co‐doped HC‐PPs‐B‐N sample has the lowest C content. The samples HC‐PPs and HC‐PPs‐N show the highest C content. For all HCs, the H content is constant at approximately 5 wt% and the O content stagnates at around 20 to 23 wt%. The ash content before and after the HTC is notable. Cabinet‐dried raw PPs have an ash content of about 8.2 wt% (Table S1 in the Supporting Information). After the HTC, the ash content of all HCs has dropped drastically to between 0.4 wt% and 1.7 wt%. It is known that the HTC process reduces the ash content by leaching of inorganic species to the liquid phase.^[28]^

PPs consist mainly of carbohydrates with a share of approximately 62 %, of which cellulose makes up 32 %, hemicellulose 10 % as well as starch 20 %.^[29]^ Moreover, PPs are composed of lignin at around 20 %, protein at about 18 %, and lipids at roughly 1 %.^[30]^

Under subcritical water conditions, the decomposition behavior of PPs differs depending on the above‐mentioned component. The acid‐catalyzed degradation process is discussed.^[31]^ This can be assumed since the pH value of the process water is in the acidic to neutral pH range (pH=4.1–7.8) after the hydrothermal syntheses are carried out (Table S2 in the Supporting Information). Under hydrothermal conditions, PPs are converted into HC via two different synthesis routes, the solved‐intermediates‐pathway and the solid‐solid‐conversion‐pathway.[^18^, ^26^, ^32^] The polysaccharides cellulose, hemicellulose, and starch are predominant via the solved‐intermediates‐pathway, whereas lignin is governed by the solid‐solid‐conversion‐pathway.[^25^, ^26^, ^32^] The polysaccharides start to hydrolyze and are cleaved into its monosaccharides.^[18]^ Via isomerization, dehydration, cyclization, and aromatization, the sugar monomers react to the intermediate 5‐hydroxymethylfurfural (HMF).^[26]^ Fragmentation of HMF results in organic acids, which further react with byproducts of the degradation process through cyclization or (poly)condensation reactions to polyfuranic chains.^[26]^ These furan chains subsequently form aromatic‐like carbon frameworks, smaller organic as well as gaseous compounds, and ultimately the solid polymeric HC is produced.^[26]^ The elemental analysis reveals a relatively high C content for all HCs between 64 wt% and 68 wt%. The carbon efficiency is in the moderate range and varies between 49 wt% and 59 wt% (Table S2 in the Supporting Information). The high C values result from the fact that mostly O‐functional structural elements are degraded.[^26^, ^31^] Besides, this might be an indication of a pronounced degree of internal condensation and cross‐linking of the polymeric carbon network of the HCs.[^26^, ^31^]

Under subcritical conditions, the PPs and the added dopants urea and B_2_O_3_ decompose. During this degradation process and the accompanying conversion to HC, a vast number of reactions take place, especially in the presence of the N precursor urea, which has already been discussed in detail elsewhere.[^25^, ^26^, ^31^] Since urea decomposes into CO_2_ and ammonia, ammonia reacts with reducing sugar (e. g. degraded hemicellulose) via Maillard reaction.^[26]^ The Maillard reaction results in the incorporation of N into the carbon framework and includes amination, imination (Schiff base), rearrangements, cyclization, degradation (e. g. Strecker degradation) reactions, or the transformation to aromatic structural motifs (e. g. pyrrole, pyrazine, furan, pyrone).[^25^, ^26^, ^31^] The non‐Maillard reactions are further essential reaction mechanisms to introduce N into the carbon network. They involve condensation (Mannich reaction), amidation (Schotten‐Baumann reaction), (reductive) amination (Leuckart‐Wallach, Eschweiler‐Clarke reaction), or ring forming reactions (Paal‐Knorr pyrrole and furan syntheses, Chichibabin pyridine synthesis or Staedel‐Rügheimer pyrazine synthesis).[^25^, ^26^, ^31^] Further cyclization reactions imply cycloadditions, which are (hetero−)Diels‐Alder reactions. These reactions contribute to the formation of N‐heterocycles.[^25^, ^26^, ^31^]

Generally speaking, N‐doping succeeded, since the N content increases. Pristine HC‐PPs indicates an N content of 3.4 wt%. Single‐doped HC‐PPs‐N exhibits the highest N content of 5.7 wt%, followed by co‐doped HC‐PPs‐B‐N with an N content of 5.5 wt%.

In contrast, B‐doping with B_2_O_3_ was ineffective under subcritical water conditions. The proportion of B that passed into the solid phase of HC‐PPs‐B‐N and HC‐PPs‐B is relatively low with values of 3.3 % and 3.7 %, respectively (Table S2 in the Supporting Information). Therefore, it can be assumed that most of the B was transferred to the liquid phase. In fact, the transition of B into the process water is roughly 71 % for HC‐PPs‐B‐N (Table S3 in the Supporting Information). The remaining 25 % could have been physisorbed B species on the carbon surface that were washed out during the filtration process with distilled water. Consequently, the assumption can be confirmed that only a very low percentage of B is covalently bonded to the carbon structure of the HC. To the best of our knowledge, studies on reaction mechanisms related to B compounds under hydrothermal conditions are still largely unknown. However, studies suggest that B structural motifs bonded in carbon scaffolds extend from substitutional B to boronic or borinic esters, and boronic acids.^[7]^ Under low‐temperature processing (<800 °C), inorganic B compounds have low doping efficiency because of their low reactivity.[^7^, ^8^, ^33^, ^34^] Instead, the use of organic B compounds might not be reasonable for they are usually highly toxic.^[33]^ Unlike N, the incorporation of B directly into the carbon matrix is fairly challenging and requires an ingenious strategy to overcome these drawbacks. The role of the dopant B_2_O_3_ during HTC remains basically unknown due to the lack of mechanistic studies. It is assumed that no major decomposition takes place during the HTC, as the B contents are very low at 0.15 wt% and 0.17 wt% for HC‐PPs‐B‐N and HC‐PPs‐B, respectively. It can be postulated that B is bonded into the carbon skeleton as follows. Once B_2_O_3_ is dissolved in water, it forms metaboric acid and boric acid (H_3_BO_3_).^[35]^ Diols (e. g. hydroxycarboxylic acids) are among the decomposition products of carbohydrates.^[31]^ As for most syntheses an acidic pH range of the process water is present, boron acids (e. g. boric or boronic acids) can undergo esterification reactions in the presence of hydroxycarboxylic acids to form boric ester (borate monoester) or boronic ester (boronate monoester).^[36]^

Importantly, in view of synthesis preparation, it is paramount that further systematic studies focus on the doping level. This should be varied and optimized. On this occasion, the dopant B_2_O_3_ is supposed to be examined more particularly. Since it has been already stated, the use of inorganic B compounds must be viewed somewhat critically due to their low doping efficiency and low reactivity.[^8^, ^33^] Indeed, it appears that B_2_O_3_ is perhaps not convenient, as a high mass balance loss was detected in this study. It has been reported that minimal B‐doping of 0.2 at% was already sufficient to enhance the specific capacitance significantly, but, it has been achieved with boric acid, H_3_BO_3_.[^37^, ^38^] Likely, the usage of a water‐soluble B compound such as H_3_BO_3_ may be more promising in the context of HTC, as it has been reported elsewhere that both a higher B content and improved specific capacitance were obtained.^[8]^ Moreover, H_3_BO_3_ might also promote the pore formation of B‐doped carbon.^[8]^ Nonetheless, it should be noted that H_3_BO_3_ is also an inorganic compound, which means that further systematic studies, e. g. with higher concentrations, may be necessary for both B_2_O_3_ and H_3_BO_3_ to assess their suitability for use.

#### 2.1.2 Thermal Behavior

**Figure 3:**
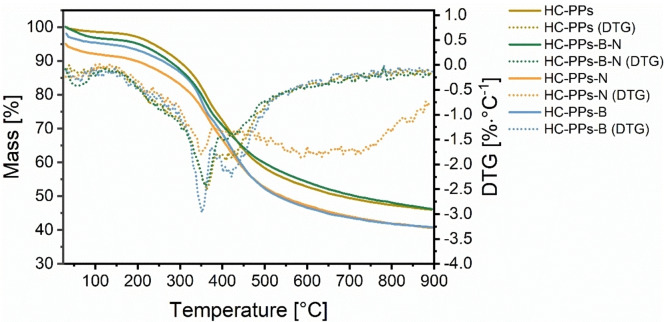
The curves of mass loss and the derivative thermogravimetry (DTG) are plotted versus the temperature of the HCs from PPs with the corresponding B and N precursors B_2_O_3_ as well as urea.

Thermogravimetric analysis (TGA) was used to determine the thermal decomposition behavior of all HCs (Figure 3).

The thermogram of all samples depicts a similar curve shape with a one‐step thermal degradation between 220 °C and 713 °C, which can be explained by the homogeneous nature of the carbonaceous matrix.[^25^, ^26^] At the beginning, the derivative TG (DTG) illustrates a very weak moisture peak at around 60 °C.[^25^, ^39^] A sharp peak is observed at 351 °C, followed by a weaker peak at 410 °C that resembles more or less a shoulder. The TG curves of both samples HC‐PPs‐N and HC‐PPs‐B display the highest mass loss of approximately 59 % over the whole temperature window. Conversely, the TG curves of the other two samples HC‐PPs and HC‐PPs‐B‐N indicate the lowest mass loss of roughly 54 % over the whole temperature range. Of all HCs, both samples are characterized by their highest thermal resistance to thermal decomposition. This can be accounted for by their higher internal degree of condensation of the polymeric carbon matrix and by their pronounced content of thermally stable N‐ and O‐structural entities, which result in a more thermally resilient HC.^[26]^ The reason for the robust network might originate from N‐structural units, N‐heterocycles (pyrrole, pyridine).[^25^, ^26^, ^31^] In addition, further N species might include graphitic/quaternary‐N and oxidized pyridine‐N.[^3^, ^40^] In the case of HC‐PPs‐B‐N from additional B functional groups.[^6^, ^7^] Various types of B‐doped functions occur bearing several kinds of structures that might be present in the carbon scaffold. This mixture of B‐, N‐ and O‐structural moieties could give this sample a stabilizing structural effect and might be a reason for the higher thermal resistance to thermal degradation compared to the other two samples HC‐PPs‐N and HC‐PPs‐B.

A HC based on lignocellulosic biomass is more prone to thermal degradation, particularly between 200 °C and 400 °C.[^26^, ^41^] This phenomenon can be confirmed in the DTG curves of the present thermal degradation pattern, especially the sharp peak at 351 °C and the shoulder‐like peak at 410 °C. In fact, within this temperature region, the main decomposition of all HCs is detected. It stands to reason that both thermolabile N‐ and O‐units are emitted.[^26^, ^31^] The absence of a further peak between 200–260 °C^[42]^ or 220–315 °C^[30]^ in the thermal decomposition pattern suggests that hemicellulose was completely degraded during HTC.

### 2.2 Characterization of Activated Carbons (ACs)

#### 2.2.1 Chemical Composition

**Figure 4:**
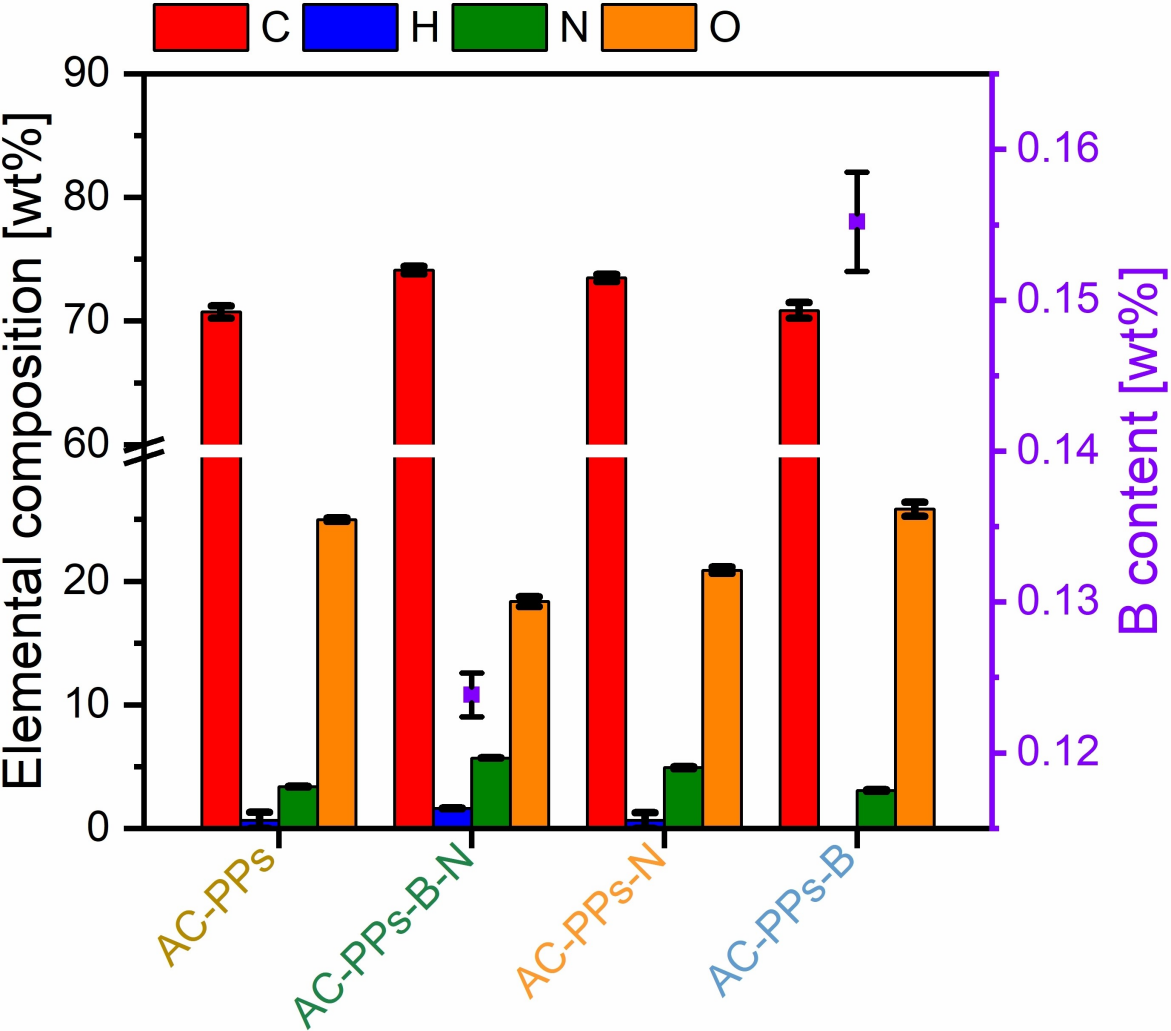
Elemental composition (columns) and B content (squares) after activation of B‐ and N‐doped HC‐PPs with ZnCl_2_. The values of the S content were not considered in the graphical plot, since the values are ≤0.3 %.

After chemical activation of all HCs with ZnCl_2_ at 900 °C, the elemental composition changes considerably (Figure 4 and Table S4 in the Supporting Information).

The chemical activation process at 900 °C is accompanied by a slight rise of the C content between 71 wt% and 74 wt% with a simultaneous drastic reduction in the H content. On the contrary, the values for the N, B, and O content remain at a stable level.

Among the samples, AC‐PPs‐B‐N represents the highest C increase to 74.1 wt%, whereas the lowest C percentage of 70.7 wt% is obtained with pristine AC‐PPs. N contents are in the range from 3.1 wt% to 5.7 wt%. Here, the highest N proportion of up to 5.7 wt% is achieved with AC‐PPs‐B‐N; the lowest N content with a value of 3.1 wt% is reached with AC‐PPs‐B. The values of the O content remain relatively constant between 18 wt% and 26 wt%, with AC‐PPs‐B‐17 having the highest percentage of 25.8 wt%. AC‐PPs‐B‐N possesses the lowest amount of O with a value of 18.4 wt%. The B content of the two samples AC‐PPs‐B‐N and AC‐PPs‐B are 0.1 wt% and 0.2 wt%, respectively. Of the chemical elements measured, only the H content shows a drastic diminution after pyrolysis. The values for the samples AC‐PPs and AC‐PPs‐B are close to zero, at (0.6±0.6) wt % and (0.7±0.7) wt %, respectively. The sample AC‐PPs‐B contains no H; only for AC‐PPs‐B‐N a stable H content of 1.6 wt% was determined. ZnCl_2_ is used as a chemical activating agent and is known to be a Lewis acid. It acts as a dehydrant leading to carbonization reactions and improves polycondensed aromatic ring reactions.[^43^, ^44^, ^45^] Further on, ZnCl_2_ reacts with water to form an acid that is corrosive and hence has a caustic effect on the carbon surface resulting in the development of the porous, channel‐like structure.[^43^, ^46^] Additionally, from the hydroaromatic skeleton, ZnCl_2_ selectively releases volatile components such as H and O in the form of H_2_O and H_2_ rather than hydrocarbons, CO, CO_2_, or oxygenated organic compounds.[^43^, ^46^, ^47^] This might be the reason for the substantial drop in the H content and at the same time the reason for the stable O content after activation, as the evolution of oxygenated substances is limited. Another possibility for the high O content may derive from thermostable O‐functional groups, e. g. phenolic, ether, and carbonyl groups, which cross‐link the polymeric carbon network imparting structural stability.[^26^, ^31^] Indeed, it should be noted here that ZnCl_2_ activation supports the formation of benzoquinone and ether functionalities.^[48]^ The volatilization frees up some sites for reactions,^[43]^ for example, cyclization reactions or annulation of ring structures to polycyclic aromatic compounds may occur.^[26]^ Likewise, thermostable N and B species may be the reason for the relatively stable N and B content after activation. N‐structural links imply quaternary‐N, pyrrole, or pyridine.[^25^, ^26^] In the case of possible B, structural connections may entail substitutional B, boronic and borinic esters, or boronic acids, which are present at 900 °C.^[7]^

#### 2.2.2 Nitrogen Adsorption‐Desorption BET Isotherms and Pore Structure Analysis

**Figure 5:**
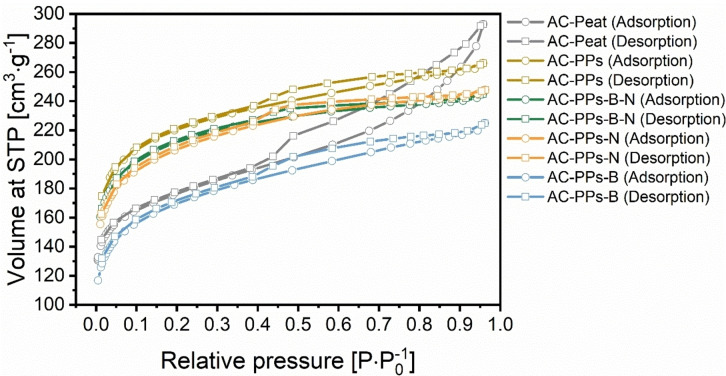
N_2_ adsorption‐desorption isotherms of the ACs based on PPs using ZnCl_2_ as an activating agent with the corresponding B as well as N precursors B_2_O_3_ and urea. The reference material is AC‐Peat.

**Figure 6:**
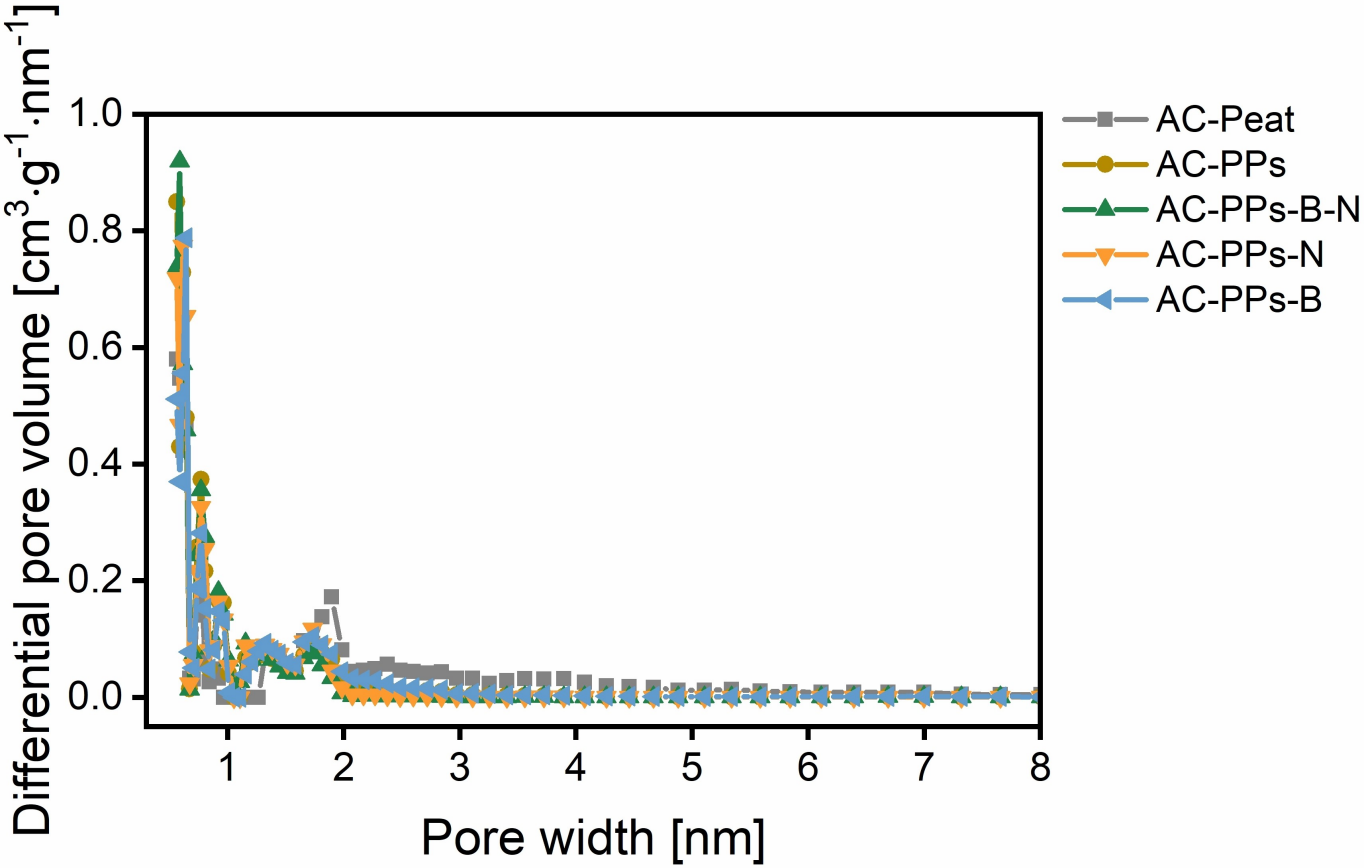
Pore size distribution calculated from N_2_ adsorption data via DFT method of pure AC‐PPs as well as the B‐ and N‐doped PPs after chemical activation with ZnCl_2_. The reference material is AC‐Peat.

To investigate the pore texture parameters such as the specific surface area (SSA), the pore size distribution, the average pore size as well as the total pore volume of all ACs, physisorption analysis utilizing N_2_ at 77 K was examined. Activated carbon made of peat (AC‐Peat) was used as reference material for the physisorption analysis. (Figure 5, Figure 6, and Table S5 in the Supporting Information).

ZnCl_2_ was utilized as a chemical activating agent. It is able to catalyze the pyrolysis of residual cellulose as well as hemicellulose in the solid.^[49]^ Hence, ZnCl_2_ is a pore‐forming agent and creates more porosity. Consequently, it enlarges the SSA.[^45^, ^49^] N_2_ sorption is suitable for studying porous materials on a wide size range of micro‐ and mesopores.^[50]^ All ACs depict high N_2_ uptakes at standard temperature and pressure (STP) on account of microporosity, presumably as a result of the elimination of volatile matter or related to the formation of (poly)condensed aromatic systems upon activation. Both phenomena cause the generation of microcavities within the pregraphitic structures.^[50]^ The results of all ACs corroborate the existence of primarily micropores (d<2 nm) in the pore size distribution (PSD) diagram (Figure 6). In the micropore scale of the PSD, for all ACs, the peaks are sharp and nearly identical. AC‐Peat exhibits a slight difference compared to the other samples at 1.9 nm. It records a comparatively sharp peak here. The BET physisorption isotherms of most ACs can be classified as type I according to the IUPAC classification criteria suggesting the presence of microporosity,[^8^, ^51^] as can be seen in the PSD diagram. For three samples, AC‐Peat, pristine AC‐PPs and AC‐PPs‐B, a combination of type I and IV isotherm can be assigned since these samples indicate not only micropores but also mesopores in the PSD diagram ranging from 2–4 nm pointing to narrow mesopores.^[8]^ For pristine AC‐PPs and AC‐PPs‐B, it is in the range of 2.0 to 3.4 nm. For AC‐Peat, it extends to a range of approximately 5.0 nm. The mesopores produced in this region are a hint that they may result from micropore collapse. This also coincides with the literature stating that mesopores obtained in the size of 2–4 nm are likely to be formed from micropore collapse.^[52]^ In these micropores (0.7 nm<d<2 nm) – this interval is also referred to as supermicropores – and in these small mesopores capillary condensation of N_2_ occurs.[^51^, ^52^] For all samples, the micropores are mostly between 0.6 and 2.0 nm in size. It is noteworthy that the share of ultramicropores (d<0.7 nm) is fairly high. For all ACs, at low relative pressure (P ⋅ P_0_^−1^) between 0.0 and 0.1, a leap in N_2_ adsorption is presented. A gradual approach to the plateau follows at higher relative pressures.^[53]^ Except in the case of AC‐Peat, there is a moderate increase within this interval. In this region, a H4 type hysteresis loop can be observed with the increment of P ⋅ P_0_^−1^ ranging from 0.39 to 0.96. According to IUPAC, the H4 hysteresis loop points to the presence of slit‐shaped micropores stemming from internal parallel pore structure.[^49^, ^54^, ^55^] Notably, ZnCl_2_ activation tends to develop a microporous structure at activation ratios, as performed in this study (HC:activator=2 : 1). It has also been published elsewhere that at low ZnCl_2_ activation ratios, a dominant micropore structure is generated.^[52]^

Additionally, the parameters of the pore texture of all ACs are listed in Table S5 in the Supporting Information. It is noticeable that the SSA values are similarly high for all samples in the interval from 627.2 to 825.3 m^2^ ⋅ g^−1^. The PSD curves denote primarily micropores and unveil a bimodal distribution indicating similar average pore size values between 0.95 and 1.38 nm, which is in agreement with the outcome of its N_2_ adsorption‐desorption isotherms.^[56]^ The total pore volumes stagnate between 0.35 and 0.45 cm^3^ ⋅ g^−1^. All AC samples are characterized by large SSAs deducing from ultramicroporosity.^[50]^ Notably, the SSA is highest for pristine AC‐PPs with a value of 825.3 m^2^ ⋅ g^−1^, an average pore size of 1.00 nm, and a total pore volume of 0.41 cm^3^ ⋅ g^−1^. Altogether, the SSAs and the total pore volumes of all ACs are comparable to the values found in the literature for B‐ and N‐doping. Only the values of the measured average pore size are larger in comparison with the literature.[^8^, ^9^] Interestingly, a decline in SSA can be monitored for the B‐ and N‐single‐doped ACs, as well as B,N‐co‐doped ACs, compared to pristine AC‐PPs. This phenomenon can be accounted for by the doping process evoking heteroatom shedding or pore blocking.^[8]^

In sum, it is apparent from the N_2_ adsorption‐desorption isotherms and the pore texture parameters that ZnCl_2_ activation succeeded in fabricating ACs with pronounced microporosity and large SSA. It is well‐known that ZnCl_2_ activation in conjunction with a biomass or char results in a microporous structure with a large SSA.^[57]^ The activation ratio (HC:activator=2 : 1) had an advantageous effect on the growth of porosity. When the obtained SSA values associated with PPs and ZnCl_2_ activation are compared with the literature, the reported SSAs of the ACs show values of 314 m^2^ ⋅ g^−1^,^[58]^ 1027 m^2^ ⋅ g^−1[56]^ up to 1078 m^2^ ⋅ g^−1^.^[59]^ The pore volumes and the total pore volumes obtained here are in the same order of magnitude as those reported elsewhere.[^8^, ^9^, ^58^] On the other hand, the previously reported values for the pore volumes demonstrate higher values with simultaneously larger SSAs. For instance, a total pore volume of 1.34 cm^3^ ⋅ g^−1^ was attained at an SSA of 1027 m^2^ ⋅ g^−1^, which is about three times higher than that reached in this study.^[56]^ This sparks the question as to why relatively low pore volumes were obtained in this study, despite larger SSAs. There may be various reasons for the low pore volumes. Mainly, parameters such as temperature, activation, or impregnation ratio play an essential role.[^56^, ^58^, ^59^] Basically, it turns out that the activation temperature has a substantial influence on the porosity development. The activation or impregnation ratio has a significant impact on the pore structure that can be controlled by varying the ratio. Low ratios result in microporosity with low pore volume, whereas high ratios lead to mesoporous ACs possessing high pore volumes.[^58^, ^59^] Additionally, chemical activation also plays an important role. The choice of feedstock and activation process are the most important parameters that have an impact on the electrochemical performance of the AC.[^10^, ^60^] The starting material exerts an influence on the porosity of the AC, whereas the PSD of the AC depends on the activation method.^[10]^ Porous AC materials with various SSAs and pore structures can be synthesized by adjusting parameters.^[48]^ For example, activation temperature, activation time, the impregnation or activation mass ratio of precursor (e. g. HC) and the chemical activating agent should be modified or a combination of two activating agents (dual activation) can be employed for future studies,[^48^, ^57^] Dual activation yields in hierarchical porous architecture combined with large SSA and a broad PSD including micropores (<2 nm), mesopores (2–50 nm), and macropores (>50 nm).[^10^, ^57^] This would considerably favor fast electrolyte ion diffusion and interactions between electrolyte and electrode by providing interconnected and short ion diffusion channels.[^11^, ^19^] Large SSA supplies abundant (electro)active sites for effective ion adsorption allowing reversible Faradaic redox reactions.^[19]^ Depending on the biomass, suitable activation and process conditions should be selected with care in order to obtain tailor‐made ACs with optimized physical‐chemical characteristics.^[48]^

Furthermore, ZnCl_2_ induces inhibitory effects on pore development and the pore properties may deteriorate at high temperatures (≥600 °C). This might be due to the sintering effect of the volatile matter with accompanying shrinkage and reorientation of the carbon structure as well as a reduced pore volume. Moreover, high temperatures might lead to the narrowing and closing of pores as well as the decrease in SSA owing to thermal shrinkage.[^46^, ^55^, ^61^] For energy storage applications, the role of pore types is controversially discussed in the literature. Commonly, it is believed that the coexistence of micro‐ and mesopores is beneficial for remarkable specific capacitances as well as high power density, since mesopores decrease the inner resistance and are conducive to fast electrolyte ion transport providing a short path for ion transport to the bulk of the electrode, which favors the development of an electric double layer (EDL).[^8^, ^9^, ^19^, ^48^, ^62^] Microporosity provides active sites for ion adsorption and is preferred to store more charges but is alone not adequate for electrode material applications,^[8,9,48.62]^ However, it has been demonstrated that entirely manufactured microporous carbons not only showed very large SSA (2967 m^2^ ⋅ g^−1^) but also outstanding electrochemical performance with excellent specific capacitance (236 F ⋅ g^−1^).^[63]^ With respect to mesopores, it has been even concluded that its presence is not necessary for fast ion diffusion, superior capacitance retention, and better power storage characteristics.[^63^, ^64^] In fact, according to the literature, the production of hierarchical porous ACs with the combination of achieving a reasonable ratio of micro‐, meso‐ and macropores are essential for very large SSA, high specific capacitances, and thus overall electrochemical performance.[^23^, ^57^, ^65^, ^66^] In addition, macroporosity acts as ion‐buffering pools (ion reservoirs) supplying a substantial ion source for ion adsorption during the charge‐discharge processes and providing free expansion spaces for the electrode.[^23^, ^52^]. It serves to lessen the mass transfer resistance of charge carriers and enables effective, short diffusion distance from mesopores to micropores for electrolyte ions,[^23^, ^48^, ^66^]

#### 2.2.3 Electrical Conductivity (EC)

**Figure 7:**
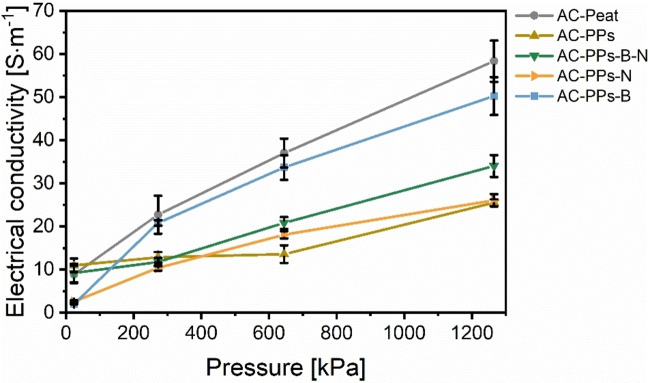
EC plotted as a function of pressure (by weights: 2, 5, and 10 kg) of the ACs of PPs utilizing ZnCl_2_ as an activating agent with the corresponding B and N precursors B_2_O_3_ and urea. The reference material AC‐Peat is denoted as a dark grey line.

The AC electrodes were applied for EC measurements. The ACs are plotted with the reference material AC‐Peat in Figure 7. The absolute values are listed in Table S6 in the Supporting Information.

The synthesized ACs in the diagram are plotted as EC as a function of pressure. The pressure is expressed by the weights 2, 5, and 10 kg and converted into the unit Pascal. The higher the pressure exerted, the higher the EC values obtained. At the maximum pressure of 1265.6 kPa, the average EC values of all ACs vary between 25.5 S ⋅ m^−1^ for pristine AC‐PPs and 50.3 S ⋅ m^−1^ for AC‐PPs‐B. These values are close to the EC value of the reference material AC‐Peat of (58.3±4.8) S ⋅ m^−1^. It is evident that the EC values of all doped ACs are higher than that of pristine AC‐PPs. The average EC value of the sample AC‐PPs‐N is only marginally higher at 26.1 S ⋅ m^−1^ than pristine AC‐PPs. In the case of the sample AC‐PPs‐B‐N, the EC value is somewhat higher at 34.0 S ⋅ m^−1^. The EC value of AC‐PPs‐B is about twice as high with a value of 50.3 S ⋅ m^−1^ compared to pristine AC‐PPs. One reason for the higher EC values in comparison with pristine AC‐PPs might be deduced from the slightly higher C content and the concurrently lower O content (Figure 4 and Table S4 in the Supporting Information). A higher C content means a more pronounced sp^2^ hybridization of C atoms in the polycondensed aromatic framework.[^26^, ^67^] In the same way, a high O content means the presence of O functionalities, which represent an electron barrier and hence lead to a diminution in EC.[^26^, ^68^, ^69^, ^70^] This means that this explanation cannot be applicable to the sample AC‐PPs‐B. In fact, the major reason for the improved average EC values compared to AC‐PPs is not only due to the doping of this one AC sample but of all ACs. Even if the N content of AC‐PPs‐B is 3.1 wt% with a B content of only 0.2 wt% and lower than that of pristine AC‐PPs (Figure 4 and Table S4 in the Supporting Information), the EC is highest with a value of 50.3 S ⋅ m^−1^. The sample AC‐PPs‐B‐N has the second highest EC value of 34.0 S ⋅ m^−1^ that is co‐doped and has the highest N content of 5.7 wt% as well as a B content of 0.1 wt%. This sample is followed by AC‐PPs‐N with the third highest EC value of 26.1 S ⋅ m^−1^ and features an N content of 4.9 wt%. N serves as an electron donor in the delocalized conjugated π electron system of the sp^2^ hybridized carbon matrix and is an n‐type dopant that boosts the EC.[^26^, ^71^, ^72^] In contrast, B acts as an electron acceptor in the conjugated π electron system and is thus a p‐type dopant. Because of the positively charged B atoms, the electrons become active and the electronic properties of the carbon ameliorate.[^5^, ^6^] The enhanced EC is the result of the combination of chemical activation and the abundance of N and B structural moieties. These include diverse N‐ and B‐active sites in the carbon lattice, which have been formed during HTC via Maillard and non‐Maillard reactions. N active sites (e. g. pyridine) trigger the formation of donor states close to the Fermi level.[^26^, ^72^] In contrast, B‐active sites (e. g. substitutional boron, boronic and borinic ester or boronic acids) result in the occurrence of acceptor states that are located above the Fermi level.[^5^, ^6^, ^7^, ^73^] The Fermi level is shifted to the conducting band changing the electronic structure.^[38]^ Apart from that, the highest EC of AC‐PPs‐B (regardless of AC‐Peat) might also be attributable to B atoms that can enhance the degree of graphitization of non‐graphitizable carbons. This is advantageous for improving the EC.^[8]^ Co‐doping of B and N contributes to preferable synergetic effects. It provides structurally complex active sites, which enable improved EC. Since the electrode AC‐PPs‐B already contains 3.1 wt% N, probably it may be the reason for improved EC. This explanation might also apply to the electrode AC‐PPs‐B‐N, which has the second highest EC of all AC electrodes (regardless of AC‐Peat). Nevertheless, the detection of such N and B structural entities was not the subject of this investigation.

Taken as a whole, the EC values of all ACs obtained here are comparatively low. In a recently published study on the synthesis of biobased N‐doped carbon electrodes, superior EC values were obtained.^[26]^ The reasons for lower EC values are thus obvious. As ACs are endowed with a pronounced porous structure, air‐filled pores might be the consequence of lower EC values.[^26^, ^74^] Factors such as the high percentage of O‐functional groups.[^26^, ^68^, ^69^, ^70^] the amorphous, non‐crystalline character.[^26^, ^71^] and the associated disordered structure.[^26^, ^68^, ^71^] of the ACs as well as deficient textural parameters.[^26^, ^71^] also play a role in resulting lower EC values.

#### 2.2.4 Electrochemical Testing

**Figure 8:**
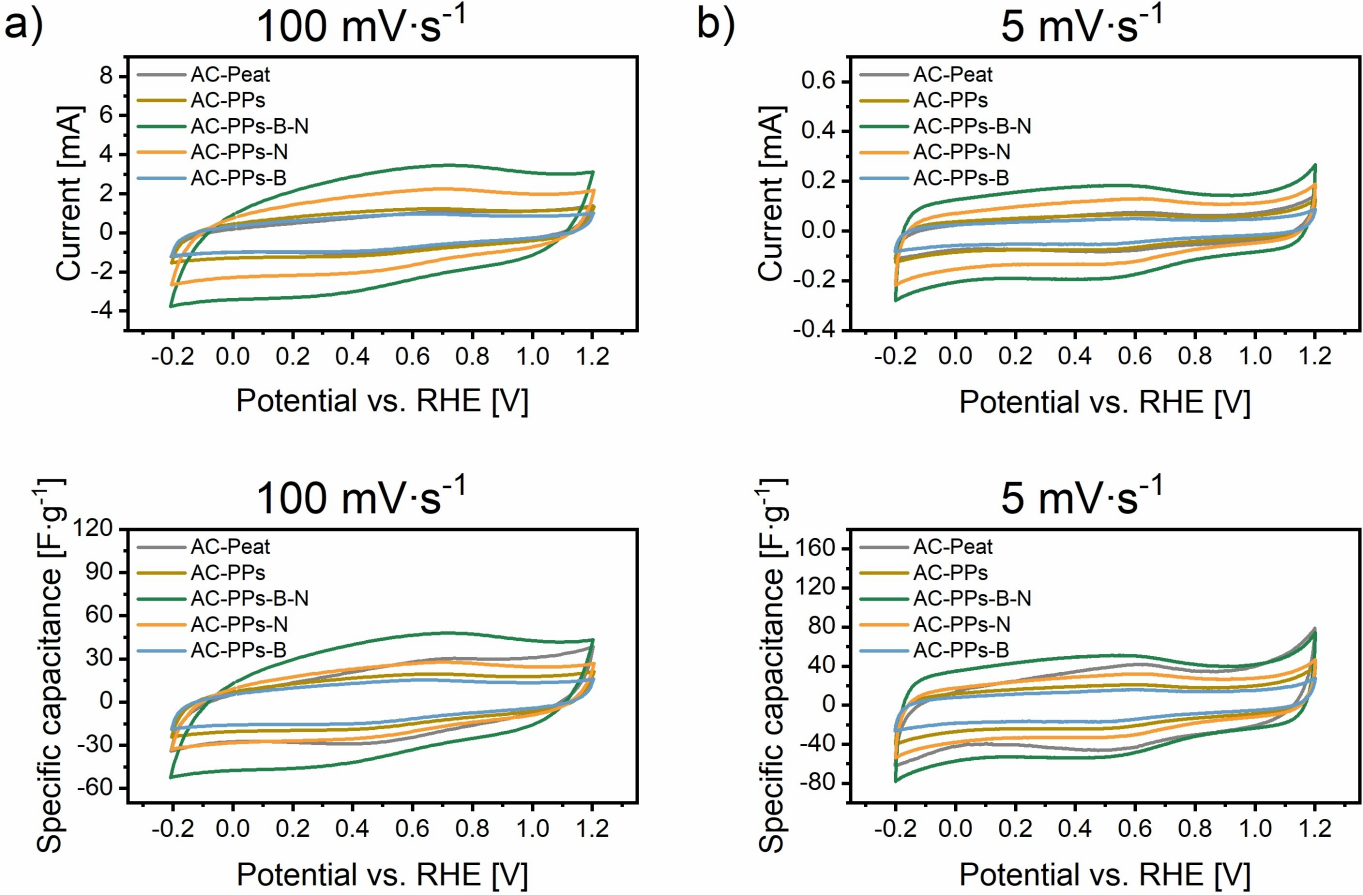
CV curves of the AC electrodes derived from PPs using ZnCl_2_ as activating agent with the corresponding B and N precursors B_2_O_3_ and urea at scan rates of (a) 100 mV ⋅ s^−1^ and (b) 5 mV ⋅ s^−1^ in 1 M H_2_SO_4_. The reference material is AC‐Peat.

Pristine AC electrode springing from PPs, the B‐ and N‐single‐doped as well as B,N‐co‐doped AC electrodes with the reference material AC‐Peat were subjected to cyclic voltammetry (CV) measurements at a higher scan rate of 100 mV ⋅ s^−1^ and lower scan rate of 5 mV ⋅ s^−1^ in 1 M H_2_SO_4_ (Figure 8, Figure S1 and Figure S2 in the Supporting Information).

The stability of the AC electrodes during the electrochemical measurement was assessed by CV over a period of approximately 50 min. The CV curves remained consistent over multiple cycles, with no significant shifts in peak positions or changes in potential or current response. Furthermore, the curves showed good overlap, indicating reproducibility and minimal degradation. The AC electrodes depict similar CV profiles and unveil a quasi‐rectangular shape within the potential range of −0.2–1.2 V. In the CV curves, anodic and cathodic half‐cycles each are endowed with a wide hump aroused by reduction as well as oxidation (redox) reactions of heteroatoms (B, N, and O) in the form of functional groups serving as active sites.[^8^, ^9^, ^26^, ^62^] The corresponding redox peaks of the anodic and cathodic half‐cycles can be identified in the following potentials (Table 1).

**Table 1:** Potentials of the redox peaks of all AC electrodes from PPs using ZnCl_2_ as activating agent with the corresponding B and N precursors B_2_O_3_ and urea at scan rates of 100 mV⋅s^−1^ and 5 mV⋅s^−1^ in 1 M H_2_SO_4_. The reference material is AC‐Peat.

	100 mV⋅s^−1^		5 mV ⋅ s^−1^	
Sample	Oxidation peak on the anodic half‐cycle [V]	Reduction peak on the cathodic half‐cycle [V]	Oxidation peak on the anodic half‐cycle [V]	Reduction peak on the cathodic half‐cycle [V]
AC‐Peat	0.73	0.41	0.61	0.51
AC‐PPs	0.68	0.41	0.61	0.50
AC‐PPs‐B‐N	0.71	0.23	0.54	0.48
AC‐PPs‐N	0.69	0.40	0.61	0.55
AC‐PPs‐B	0.64	0.41	0.60	0.49

**Figure 9:**
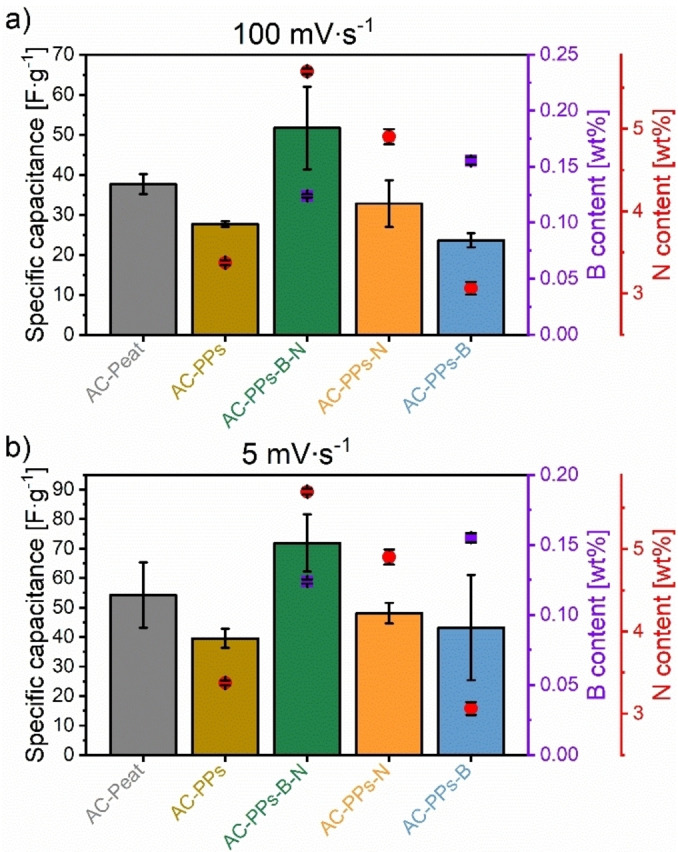
Specific capacitances of the AC electrodes based on PPs activated with ZnCl_2_ as activating agent and the corresponding B as well as N precursors B_2_O_3_ and urea at a scan rate of (a) 100 mV ⋅ s^−1^ and (b) 5 mV ⋅ s^−1^ in 1 M H_2_SO_4_. The reference material is AC‐Peat.

It is apparent from Figure 8 that these half‐cycles are more intense at a lower scan rate as higher specific capacitances are achieved. Conversely, lower specific capacitances are obtained at a higher scan rate (Figure 9 and Table S7 in the Supporting Information). The increasing scan rate not only transforms the shape of the CV profiles towards a distortion with a slight incline but also reduces the values of the specific capacitances.^[26]^ At 5 mV ⋅ s^−1^, both the CV curves are less distorted and the specific capacitances are higher. This means that more mobile charge carriers (e. g. ions or electrons) diffuse easily into the narrow pore sizes of the carbon network of the active AC electrodes.[^9^, ^26^, ^75^] The average pore size of all ACs ranges from 0.95–1.38 nm and is larger than that of the dissolved electrolyte ions SO_4_^2−^ (5.33 Å)>K^+^≈H_3_O^+^ (3.62–4.2 Å).[^9^, ^19^, ^75^] As a result, the mobile transport of these charge carriers within the pore system is advantaged and enables higher physical charge storage through more extensive electrostatic interactions and physisorption. Thereby, the resulting EDL at the electrode/electrolyte interface (EEI) is more pronounced.[^9^, ^26^]

At a high and low scan rate of 100 mV s^−1^ and 5 mV ⋅ s^−1^, the electrode AC‐B‐N achieves the highest average specific capacitance of 51.7 F ⋅ g^−1^ and 71.9 F ⋅ g^−1^, respectively. Although the standard deviation is relatively high, the determined specific capacitances exceed those of the reference material AC‐Peat at 100 mV ⋅ s^−1^ (37.7±2.5 F ⋅ g^−1^) and at 5 mV ⋅ s^−1^ (54.3±11.0 F ⋅ g^−1^). The electrode AC‐N reveals the third highest values of the average specific capacitances both at 100 mV⋅s^−1^ and at 5 mV⋅s^−1^ of 32.8 F⋅g^−1^ and 48.1 F⋅g^−1^, respectively. Pristine AC‐PPs possesses the fourth highest value of average specific capacitance of 27.7 F ⋅ g^−1^ at 100 mV ⋅ s^−1^; at 5 mV ⋅ s^−1^, the calculated average specific capacitance results in a value of 39.5 F ⋅ g^−1^ and is consequently the lowest compared to all other ACs. The electrode AC‐B gives the lowest average specific capacitance of 23.6 F ⋅ g^−1^ at 100 mV ⋅ s^−1^. At 5 mV ⋅ s^−1^, the value of the specific capacitance is 43.3 F ⋅ g^−1^ and is thus higher than that of pristine AC‐PPs (39.5 F ⋅ g^−1^), but the standard deviation is relatively large. Despite the lower average specific capacitance of AC‐PPs‐B, it is obvious that the B‐ and N‐single‐doped ACs feature higher specific capacitances. The B,N‐co‐doped electrode even shows the highest specific capacitance of all measured ACs at high and low scan rates and outperforms the reference material AC‐Peat. The highest specific capacitances of AC‐PPs‐B‐N are ascribable to the highest N content of 5.7 wt%, a minimal B proportion of 0.1 wt%, a large SSA of 793.5 m^2^ ⋅ g^−1^ with a median pore size of 0.95 nm and an increased EC of 34 S ⋅ m^−1^. Despite the comparatively lower average pore size, the beneficial combination of the heteroatoms B and N changes the acid/base or electron donor/acceptor characteristics and facilitates the access for charge carrier transport of the electrolyte inside the active porous carbonaceous electrode.[^8^, ^9^, ^26^] The B,N‐co‐doped electrode alters the (surface) polarity of the carbon scaffold and the hydrophilic nature ameliorates the surface wettability of the electrode in the electrolyte allowing it to enter the ions into micropores in a simple way.[^8^, ^9^] These synergistic effects promote the ion kinetics enhancing the ion transfer efficiency^[26]^ and simplifying the electron transfer ability.^[8]^ Since the dissolved electrolyte ions possess a small ionic radius, they diffuse into the hierarchical pores systems relatively unhindered, which means that the charge carrier transport inside the carbon electrode and at the EEI is favored.[^9^, ^26^, ^62^] On account of the smaller ionic radii, it enables the ion center to come closer to the carbon electrode surface yielding an increased capacitive performance.^[9]^ These properties are predestined for charge storage mechanisms at the EE.[^26^, ^62^] It is evident from Figure 8 that the O content also plays a significant role in the capacitive performance. Figure 4 and Table S4 in the Supporting Information display an O share between 18.4 and 25.8 wt%. B‐ and N‐derived functions as well as O‐functional groups are involved in pseudocapacitive behavior through basic surface structures. In acidic electrolyte, they contribute actively to reversible Faradaic redox reactions.[^8^, ^9^, ^26^, ^62^, ^76^] In terms of N‐structural elements, pyridine, pyrrole, and quaternary‐N are active sites.^[76]^ Pyridinic‐N and pyrrolic‐N are bonded at the edge sites of the graphene layer and induce pseudocapacitance,[^26^, ^76^] whereas quaternary‐N ameliorates the electron transfer by providing its lone pair and hence improves the electron donor propertie.[^26^, ^76^] Concerning B‐active sites, the detailed mechanism of the Faradaic redox reactions is not yet fully elucidated.^[27]^ Having said that, the existence of B‐ and/or O‐functional groups might be involved in reversible redox reactions.[^27^, ^38^, ^77^] B‐active sites may include substitutional boron, boronic and borinic ester, or boronic acids.[^5^, ^7^] They are electron‐deficient, draw the electrons and boost the electron acceptor characteristics.[^3^, ^5^, ^6^] With regard to O‐active sites, benzoquinone structures and ether groups might exist, since they are preferably formed by using the chemical activating agent ZnCl_2_.^[48]^ Generally, B‐, N‐ and O‐structural entities embedded in the carbon skeleton modify the polarity of the electrode surface and strengthen the surface wettability at the EEI giving rise to pseudocapacitive effects as well as improving the rate performance.[^8^, ^9^, ^26^, ^48^, ^62^, ^76^] As a result, the charge carriers preferentially migrate through the pore system to the electrode surface provoking an increased specific capacitance.[^8^, ^26^, ^62^] Considering the pore textural parameters (Table S5 in the Supporting Information), AC‐PPs‐B‐N features an SSA of 793.5 m^2^ ⋅ g^−1^, an average pore size of 0.95 nm, and a total pore volume of 0.38 cm^3^ ⋅ g^−1^. It has been stated that a microporous system is present (Figure 6). As already indicated, the pore textural parameters are not the best‐developed in comparison with the literature. Hence, it can be assumed that the active sites consisting of B, N, and O, which are incorporated in the carbon network, play a key role in diffusing ions or electrons through the porous system to the active carbon surface. This diffusion may occur beneficially and redox reactions are facilitated that eventually induce an increase in the overall capacitive performance.[^8^, ^9^, ^26^] The synergistic effects of B‐, N‐ and O‐structural moieties are consequently crucial for the development of the electric double‐layer capacitance (EDLC) and pseudocapacitance.[^8^, ^9^, ^26^, ^62^] Importantly, with a meaningful amount of O content, the positive effects accompanied by O‐functional groups, such as an enhancement in surface wetting or pseudocapacitance, are apparent.[^9^, ^26^, ^62^] Yet, the opposite can occur if the O content exceeds a certain level. For example, the deviation of the typical rectangular profiles of the cyclic voltammograms (Figure 8) is related to O‐functionalities on the carbon electrode surface.[^26^, ^78^, ^79^] Other reasons are the relatively low EC values and high intrinsic resistance that can be associated with slow electron‐transfer rates.[^26^, ^74^] The resistance is also incurred by the limited mobility of the charge carriers in the microporous system.[^26^, ^78^] Moreover, O‐containing functional groups evoke a high electron resistance and function as an electron transfer barrier.[^26^, ^69^] This phenomenon seems to apply to all AC electrodes, but especially to pristine AC‐PPs. This sample is characterized by the highest O content of 25.0 wt% (Figure 4 and Table S4 in the Supporting Information). This high O content appears to have a negative impact on the physical‐chemical properties, as the pristine AC‐PPs electrode not only has the lowest EC value of all the ACs but also the lowest specific capacitance at 5 mV ⋅ s^−1^. In the cyclic voltammograms, it is noteworthy that the redox peaks are little but visible. The reasons for the lack of pronounced peaks are due to Faradaic charge transfer mechanisms, which encompass a large variety of reactions with numerous onset potentials.[^26^, ^80^] There can be no question of a single reaction since the mere existence of N‐structural entities in the carbon matrix along with the protons in the acidic electrolyte can enter into a multitude of reactions.[^26^, ^80^, ^81^]

The results of the physical‐chemical measurements obtained here were compared with the literature. PPs‐based AC electrodes were analyzed in various studies (Table S8 in the Supporting Information). The values of the SSAs are between 103 and 1911 m^2^ ⋅ g^−1^. The specific capacitance values range from 61 to 603 F ⋅ g^−1^ in acidic electrolyte. In a neutral environment, specific capacitances of 98 to 323 m^2^ ⋅ g^−1^ were achieved.

The SSAs of 627 to 825 m^2^ ⋅ g^−1^ obtained in this investigation correlate with the literature values. Nevertheless, the highest specific capacitances achieved here with a value of 51.7 F ⋅ g^−1^ at 100 mV ⋅ s^−1^ and 71.9 F ⋅ g^−1^ at 5 mV ⋅ s^−1^ do not quite match the literature values. This means that the strategy for producing PPs‐derived AC electrodes still offers plenty of room for optimization. It should be highlighted that a very large SSA does not necessarily go hand in hand with a very high specific capacitance. It is rather the opposite case. With a comparatively low SSA of 314 m^2^ ⋅ g^−1^, the highest specific capacitance of 603 F ⋅ g^−1^ was determined. Another important reason for the low specific capacitances might be the inhomogeneous distribution of the slurry on the glassy carbon (GC) surface of the electrode tip. This might affect the regular distribution of physisorbed charge carriers at the EEI and finally may lead to an impairment of the EDL.^[26]^

To summarize, the AC‐PPs‐B‐N electrode indicates beneficial physical‐chemical properties, which are also reflected in the electrochemical measurements. Both at a high and low scan rate of 100 mV s^−1^ and 5 mV ⋅ s^−1^, the electrode gives the highest average specific capacitance of 51.7 F ⋅ g^−1^ and 71.9 F ⋅ g^−1^, respectively. In terms of chemical composition, AC‐PPs‐B‐N has a maximum N content of 5.7 wt%, a minimum B content of only 0.1 wt%, and a comparatively low O content of 18.4 wt% compared to the other electrodes. Additionally, this electrode exhibits a large SSA of 793.5 m^2^ ⋅ g^−1^ and an increased EC value of 34 S ⋅ m^−1^. All these combined effects contribute to an improvement in the material properties and ensure, among other things, that the charge carrier transport within the pore system and the charge storage processes of the electrode as a whole are considerably enhanced. Similarly, the synergetic effects of B‐, N‐ and O‐derived active sites are of great relevance for the formation of the EDLC and pseudocapacitance, since they serve as active sites. All of these B‐, N‐, and O‐functions are bonded in the polymeric carbonaceous framework and implicated in Faradaic redox reactions that are advantageous for pseudocapacitive behavior. Despite these initial positive electrochemical measurements, the results of the physical‐chemical material properties presented here still need to be optimized to be more convenient for supercapacitor application. Indeed, efforts should be made to adjust the ratio of micro‐ and mesopores in order to ameliorate the specific capacitance. Besides, special attention should also be paid to synthesis preparation. On this occasion, the doping level should be tailored and the HC‐to‐impregnation or activation ratio optimized. Equally, care should be taken to guarantee a homogeneous distribution of the slurry on the GC surface of the electrode tip of the electrochemical cell in order to receive reproducible data and avoid falsified, invalid specific capacitance values.

## 3 Conclusions

In this work, a simple, cost‐effective, and efficient synthesis route has been employed to fabricate porous single B‐ and N‐ as well as B,N‐co‐doped AC electrodes using PPs as waste biomass. Via HTC, the “in situ doping” of PPs with the N‐dopant urea has succeeded at mild reaction conditions. These raw materials have undergone plenty of Maillard and non‐Maillard reactions transforming the HC into a structurally compact cross‐linked scaffold with a large variety of chemically surface‐reactive O‐ and N‐ structural motifs. The B‐dopant B_2_O_3_ reveals a low reactivity under subcritical conditions. A small percentage of about 3 % is incorporated into the carbon lattice via esterification reactions. Hence, it features a low doping efficiency. Upon chemical activation with ZnCl_2_, the ACs demonstrate a primarily microporous structure, with the co‐doped AC‐PPs‐B‐N exhibiting a large SSA with a value of 793.5 m^2^ ⋅ g^−1^. The electrodes AC‐PPs‐B,N as well as AC‐PPs‐N reach maximum N content of 5.7 wt% and 4.9 wt%, respectively. The B content for AC‐PPs‐B‐N and AC‐PPs‐B amounts to 0.1 wt% and 0.2 wt%, respectively. B‐ and N‐doping influence the physical‐chemical characteristics of the AC electrodes. Energy storage application displays improved electrochemical performance for the electrode AC‐PPs‐B‐N with a superior specific capacitance of 51.7 F ⋅ g^−1^ at a higher scan rate (100 mV ⋅ s^−1^) and of 71.9 F ⋅ g^−1^ at a lower scan rate (5 mV ⋅ s^−1^). These values even surpass those of the reference material AC‐Peat, which indicates that B,N‐co‐doping exerts a beneficial impact on the specific capacitance due to synergetic effects, especially pseudocapacitance behavior.

## Experimental Section

### Materials

Waxy potatoes from Germany of the “Bernina” variety were purchased from a local supermarket in Harthausen, Germany. The potatoes were peeled and these peels were dried overnight in a drying cabinet at 105 °C. After drying, the PPs were ground with a laboratory mill (IKA MultiDrive control, Staufen, Germany) for 30 sec each at 3000 rpm, 6000 rpm, and 11000 rpm to achieve a homogeneous material for the syntheses. Boric anhydride (puriss. p.a., ≥98 %) was supplied by Sigma Aldrich (Merck KGaA, Darmstadt, Germany). Urea (high purity, ≥99.5 %) was obtained from VWR Chemicals (Solon, Ohio, USA). Zinc chloride (AnalaR NORMAPUR) was purchased from VWR Chemicals (Leuven, Belgium). Hydrochloric acid (EMSURE^®^, fuming 37 %, for analysis) was supplied by Merck KGaA (Darmstadt, Germany). Ethanol (analytical standard, 99 %), polytetrafluoroethylene (PTFE), and sulfuric acid (Suprapur, 96 %) were purchased from Sigma Aldrich (Merck KGaA, Darmstadt, Germany). Carbon black (acetylene, 100 % compressed, 99.9+ %) was obtained by Alfa Aesar (ThermoFisher GmbH, Kandel, Germany). The reference material Norit^®^ PK 1–3, from peat, steam activated, granular (particle size: 1–3 mm) was obtained from Norit Nederland B.V. (Amersfoort, The Netherlands). All chemicals were of analytical grade and used as received without further purification.

### Hydrothermal Synthesis of B‐ and N‐Single‐doped as well as B,N‐Co‐doped Carbon Materials

Finely ground PPs as a carbon source, B_2_O_3_, and urea as B and N precursors, respectively were utilized as raw materials for the HTC. For the hydrothermal syntheses, the following samples were prepared in distilled water (150 mL). In a series of experiments, PPs (23.84 g) and a B_2_O_3_ suspension (0.66 wt%, 1.16 g, 16.7 mmol), PPs (24 g) and a urea solution (0.57 wt%, 1.00 g, 16.7 mmol), PPs (22.84 g), B_2_O_3_ (0.66 wt%, 1.16 g, 16.7 mmol) in combination with urea (0.57 wt%, 1.00 g, 16.7 mmol) were mixed. Pristine PPs (25 g) were prepared for comparison. The reaction mixtures were added to stainless‐steel autoclaves (250 mL volume). Afterwards, the firmly sealed autoclaves were placed in a temperature‐programmable gas chromatography (GC) oven and heated to 220 °C within about 40–60 min. After 2 h of reaction time, the autoclaves were cooled down to room temperature in cold water. Subsequently, the autoclaves were opened and the suspensions were vacuum‐filtered (qualitative filter paper, 413, VWR, Leuven, Belgium). Before the filtration process with distilled water, the filtrates (process water) were collected separately for further analysis. Thereupon, the filter cakes were washed multiple times with distilled water (2–3 L) to approximate pH neutrality (pH=6.5–7.1). The HCs were dried overnight in a drying cabinet at 105 °C. Finally, the HCs were ground into powder and homogenized in a laboratory mill (30 sec, 3000 rpm) from IKA^®^‐Werke GmbH & Co. KG (Staufen, Germany). Each HC was synthesized in duplicate.

The HC yields were calculated according to equation 1:

(1) $$HC\;Yield\;\left[\%\right]=\frac{Mass\;of\;obtained\;solid\;after\;HTC\;\left[g\right]}{Initial\;mass\;of\;reactants\;\left[g\right]}•100$$

The carbon efficiency was determined following formula 2:

(2) $$Carbon\;efficiency\;\left[\%\right]=\frac{Mass\;of\;carbon\;in\;the\;HC\;after\;HTC\;\left[g\right]}{Initial\;mass\;of\;carbon\;in\;the\;starting\;materials\;\left[g\right]}•100$$

### Chemical Activation

The HCs were chemically activated with the activating agent ZnCl_2_. The chemical activator was blended with the HC at a mixing ratio of HC:activator=2 : 1 and triturated intensively in an agate mortar. Afterwards, the HC/ZnCl_2_ mixtures were transferred to stainless‐steel crucibles. The crucibles were then placed in a batch reactor and introduced into the muffle furnace, which was heated to 800 °C at a heating rate of 10 °C⋅min^−1^ under an inert atmosphere with a constant N_2_ flow of 7.5 NL⋅min^−1^ at an N_2_ pressure of 1.5 bar. Once 800 °C was reached, the residence time was 1 h. After activation, the reactor with the crucibles inside was removed and continued to be flushed with N_2_ for 1 h to cool to room temperature. The ACs were then carefully mixed with 1 M HCl (125 mL) and the mixtures were washed for 1 h with stirring to remove residues of the ZnCl_2_ activating agent. Thereafter, the suspensions were vacuum‐filtered (qualitative filter paper, 413, VWR, Leuven, Belgium) and washed several times with distilled water (4–6 L) until approximate pH neutrality (pH=6.4–6.8) was achieved. Finally, the ACs were dried overnight in a drying cabinet at 105 °C. Each AC was produced in duplicate. In the diagrams, the values are represented as mean and the standard deviation as error bars.

### Characterization

#### Elemental Analysis

The elemental compositions of the PPs and the chars were determined by elemental analysis operating a Euro EA‐CHNS analyzer, 3000 Series (HEKAtech GmbH, Wegberg, Germany) according to DIN standard 51732. For B‐containing HCs and ACs, the O content was calculated by subtraction using equation 3:

(3) $$O\;\left[\%\right]=100^{\%}-C\left[\%\right]-H\left[\%\right]-N\left[\%\right]-S\left[\%\right]-B\left[\%\right]-Ash\left[\%\right]$$

The elemental analysis was carried out in duplicate. In the diagrams, the values are represented as mean and the standard deviation as error bars.

#### Ash Content

The ash content in the PPs and in the HCs was determined in accordance with DIN EN 14775 and DIN EN 51719, respectively. The ash content was determined in duplicate. In the diagrams, the values are displayed as mean and the standard deviation as error bars.

#### Thermogravimetric Analysis (TGA)

TG analyses were measured with the instrument NETZSCH STA Jupiter^®^ 449 F5 (NETZSCH‐Gerätebau GmbH, Selb, Germany). For the measurements, corundum crucibles (85 μL volume, NETZSCH‐Gerätebau GmbH, Selb, Germany) were filled with varying HC masses depending on the powder density between 5 and 20 mg. The HCs were flushed with a constant N_2_ volumetric flow rate of 50 mL⋅min^−1^ as well as 20 mL⋅min^−1^ of N_2_ as a protective gas and heated up to 900 °C with a heating rate of 10 K⋅min^−1^.

#### Inductively Coupled Plasma Optical Emission Spectroscopy (ICP‐OES)

All B‐containing HCs and ACs as well as B‐containing liquid samples were characterized externally by ICP‐OES at the Core Facility Hohenheim (Emil‐Wolff‐Str. 12, 70599 Stuttgart, Germany). The B content of samples was determined using ICP‐OES after thermal decomposition in acid. In short, about 500 mg of the sample was weighed accurately into glass tubes. 2 mL of HNO_3_ was added, and the tubes were filled with double‐distilled water to a final volume of 10 mL and vortexed to ensure sample homogeneity. Following this, microwave digestion was performed using an Ultra Clave III (MLS Mikrowellen‐Labor‐Systeme GmbH, Leutkirch, Germany), in which the temperature was gradually increased from 80 to 200 °C at 900 W and 100 bar. The samples were cooled down and diluted adequately for ICP‐OES analysis (Agilent 5110, Santa Clara, California, USA). For calibration, an ICP multi‐element standard solution (Inorganic Ventures, Christiansburg, Virginia, USA) was used in concentrations from 0.01 up to 100 mg⋅L^−1^, prepared with double‐distilled water and HNO_3_. The B content was determined in duplicate. In the diagrams, the values are indicated as mean and the standard deviation as error bars.

#### Brunauer‐Emmett‐Teller (BET) – Specific Surface Area (SSA)

The evaluation of the SSA of ACs was carried out by applying N_2_ as an adsorptive. The adsorption and desorption isotherms are examined using the NOVA 4000e from 3P Instruments GmbH (Odelzhausen, Germany). Before the measurement, the AC is degassed between 12 and 24 h at 180 °C. Afterwards, the measurement is performed with liquid N_2_ as a coolant at −196 °C for N_2_ adsorption in the pores of the char. When the pressure is raised, adsorbed N_2_ is emitted that yields in the pressure‐dependent adsorption and desorption isotherms. From this, the specific BET surface area is determined as follows:

(4) $$q=\frac{K•q_{max}•C_{eq}}{\left(C_{sat}-C_{eq}\right)•\left(1+\frac{\left(k-1\right)•C_{eq}}{C_{sat}}\right)}$$

With *q*=loading of the adsorbent, *K*=adsorption coefficient, *q_max_* =maximum concentration of the adsorbate in a monolayer, *C_eq_* =concentration of the adsorbate in the solution, C_sat_=solubility of the adsorbate.

#### Electrical Conductivity (EC)

The EC of the ACs was assessed by adopting the technique described elsewhere.^[82]^ The ACs were filled into an acrylic glass cylinder attached to a brass base. The AC powder sample was compressed by the upper brass plunger. The electrical resistance was detected by placing three different weights (2, 5, and 10 kg) successively on the upper brass plunger (0.192 kg). The following equation 5 was applied to calculate the weight:

(5) $$F_{g}=\left(m_{dw}\left(brass\;plunger\right)+m\right)•g$$

Where *F_g_* =weight [N], *m_dw_(brass plunger)*=dead weight of the brass plunger [0.192 kg], *m*=mass [kg], *g*=local acceleration of free fall [9.81 m⋅s^−2^].

The force of the three various weights acting perpendicular to the carbon surface is utilized to determine the pressure:

(6) $$p=\frac{F_{g}}{A}$$

With *p*=pressure [Pa], *F_g_* =weight [N], *A*=area of the brass base [7.9 ⋅ 10^−5^ m^2^].

Finally, the EC of all ACs was calculated according to formula 7:

(7) $$\sigma =\frac{h}{R•A}$$

Where *σ*=electrical conductivity [S⋅m^−1^], *h*=height of the sample [m], *R*=electrical resistance [Ω], *A*= area of the brass base [7.9 ⋅ 10^−5^ m^2^].

The EC measurements were conducted in triplicate. In the diagrams, the values are shown as mean and the standard deviations as error bars.

#### Electrochemical Analysis – Cyclic Voltammetry (CV)

Electrochemical analyses were conducted in a standard three‐electrode cell using a PGSTAT204 potentiostat from Metrohm GmbH (Filderstadt, Germany). The working electrode (WE) has been prepared from a mixture of AC and carbon black in a weight ratio of 9 : 1 (67.5 mg:7.5 mg). The heterogeneous solid mixture was dispersed in ethanol (1.5 mL) and polytetrafluoroethylene (PTFE) (10 μL). The slurry was mixed for about 30–60 sec by means of a vortex mixer (Vortexer, Reax top) from Heidolph Instruments GmbH (Schwabach, Germany). Afterwards, the slurry (4 μL) was dropped on an electrode tip (diameter of the electrode disc: 3 mm) made of glassy carbon (GC) with a shaft material consisting of polyether ether ketone (PEEK) from Metrohm GmbH (Filderstadt, Germany). To ensure the reproducibility of the measuring results, the contaminated GC surface of the electrode tip was regenerated after each electrochemical measurement by mechanical cleaning with the help of a polishing set with the finest aluminum oxide powder (grain size: 0.3 μm) from Metrohm GmbH (Filderstadt, Germany).

The measurements were performed in H_2_SO_4_ (1 M, 125 mL) electrolyte with a Pt sheet from Metrohm GmbH (Filderstadt, Germany) as a counter electrode (CE) and a reversible hydrogen electrode (HydroFlex^®^, RHE) from Gaskatel GmbH (Kassel, Germany) as a reference electrode (RE). Before starting the measurement, the electrochemical cell was flushed with N_2_ for 5 min. The CV measurements were recorded in a potential window of −0.2–1.2 V with scan rates of 5 mV ⋅ s^−1^ and 100 mV ⋅ s^−1^ applying NOVA 2.1.4 software from Metrohm GmbH (Filderstadt, Germany). The specific capacitance was determined by the following equation 8:

(8) $$C=\frac{1}{m•\nu •\Delta V}\int_{V_{i}}^{V_{f}}I•VdV$$

Where *m*=mass of the AC [mg], *ν*=scan rate [mV ⋅ s^−1^], *ΔV*=voltage interval [V], $\int_{V_{i}}^{V_{f}}I\left(V\right)dV$ =integral of the current as a function of the voltage between the voltage limits *V_i_* and *V_f_* [V⋅A].

Each electrochemical measurement lasted an average of 50 min and was carried out in duplicate. In the diagrams, the specific capacitance values are illustrated as mean and the standard deviations as error bars.

The area of a cyclic voltammogram was determined by using Origin software (OriginPro 2020, OriginLab Corporation).

- Biomass
- Doping
- Hydrothermal synthesis
- Activation
- Carbon electrode
- Electrochemistry

## Conflict of Interests

The authors declare no conflict of interest.

4

## Supporting information

As a service to our authors and readers, this journal provides supporting information supplied by the authors. Such materials are peer reviewed and may be re‐organized for online delivery, but are not copy‐edited or typeset. Technical support issues arising from supporting information (other than missing files) should be addressed to the authors.

Supporting Information

## Data Availability

The data that support the findings of this study are available from the corresponding author upon reasonable request.
