# Supplementary material for: B,N‐Doped Activated Carbon‐Based Electrodes from Potato Peels for Energy Storage Applications
Source: ChemistryOpen. 2025 Feb 19;14(3):e202400527. doi: 10.1002/open.202400527 (PMC12128159; doi:10.1002/open.202400527)
Supplement: Supplementary file 1 — Supporting Information [file OPEN-14-e202400527-s001.pdf]

ChemistryOpen

Supporting Information

# B,N-Doped Activated Carbon-Based Electrodes from Potato Peels for Energy Storage Applications

Jan Willem Straten,* Muhammad-Jamal Alhnidi, Ghassan Alchoumari, Krishna Sangam, and Andrea Kruse

# Supporting Information

## B,N-Doped Activated Carbon-Based Electrodes from Potato Peels for Energy Storage Applications

Jan Willem Straten,*[a] Muhammad-Jamal Alhnidi,[a] Ghassan Alchoumari,[a] Krishna Sangam,[a] and Andrea Kruse[a]

[a] Dr. J. W. Straten, Dr. M.-J. Alhnidi, G. Alchoumari, K. Sangam, Prof. Dr. A. Kruse
University of Hohenheim, Institute of Agricultural Engineering, Department of Conversion Technologies of Biobased Resources,
Garbenstr. 9, 70599 Stuttgart, Germany
E-mail: jan.straten@uni-hohenheim.de, jan.straten1@gmail.com

Table S1: Elemental composition ash content of the starting material PPs and its HC with the corresponding B and N precursors $B_2O_3$ and urea.

| Sample | C [wt%] | H [wt%] | N [wt%] | S [wt%] | B [wt%] | Ash [wt%] | O [wt%] |
|---|---|---|---|---|---|---|---|
| PPs | 42.7±0.4 | 5.9±0.03 | 2.4±0.03 | 0.09±0.01 | n/a | 8.2±0.06 | 40.7±0.4 |
| HC-PPs | 67.8±0.8 | 5.9±0.2 | 3.4±0.05 | 0.06±0.002 | n/a | 0.4±0.0 | 22.4±1.1 |
| HC-PPs-B-N | 64.4±0.05 | 5.4±0.05 | 5.5±0.6 | 0.02±0.02 | 0.15±0.001 | 1.7±0.1 | 22.8±0.5 |
| HC-PPs-N | 67.5±0.6 | 5.8±0.01 | 5.7±0.2 | 0.02±0.02 | n/a | 0.8±0.1 | 20.2±1.0 |
| HC-PPs-B | 66.6±0.5 | 5.7±0.05 | 3.9±0.1 | 0.07±0.01 | 0.17±0.001 | 0.6±0.05 | 22.9±0.5 |

Table S2: HC yield, carbon efficiency, mass balance of B in HC, and pH value of the HCs stemming from PPs with the corresponding B and N precursors $B_2O_3$ and urea.

| Sample | HC yield [%] | Carbon efficiency [%] | Mass balance of B in HC [%] | pH |
|---|---|---|---|---|
| HC-PPs | 36.9±0.1 | 58.6±0.9 | n/a | 5.5±0.3 |
| HC-PPs-B-N | 32.8±0.1 | 53.0±0.2 | 3.3±0.001 | 5.6±0.05 |
| HC-PPs-N | 31.3±1.4 | 50.5±1.9 | n/a | 7.8±0.2 |
| HC-PPs-B | 30.1±1.2 | 49.2±1.6 | 3.7±0.001 | 4.1±0.06 |

Table S3: B content of the process water and mass balance of B in the process water (PW) obtained after HTC.

| Sample | B [wt%] | Mass balance of B in process water [%] |
|---|---|---|
| PW-B-N | 0.17±0.00 | 70.67±0.00 |
| PW-B | 0.38±0.00 | - |

Table S4: Elemental composition of the ACs of PPs utilizing $ZnCl_2$ as activating agent with the corresponding B and N precursors $B_2O_3$ and urea.

| Sample | C [wt%] | H [wt%] | N [wt%] | S [wt%] | B [wt%] | O [wt%] |
|---|---|---|---|---|---|---|
| AC-PPs | 70.7±0.5 | 0.7±0.7 | 3.4±0.03 | 0.3±0.01 | n/a | 25.0±0.1 |
| AC-PPs-B-N | 74.1±0.3 | 1.6±0.04 | 5.7±0.03 | 0.08±0.004 | 0.1±0.0 | 18.4±0.4 |
| AC-PPs-N | 73.5±0.3 | 0.6±0.6 | 4.9±0.1 | 0.09±0.005 | n/a | 20.9±0.3 |
| AC-PPs-B | 70.9±0.6 | 0.0±0.0 | 3.1±0.08 | 0.09±0.003 | 0.2±0.0 | 25.8±0.6 |

Table S5: Pore textural parameters involving specific surface area (SSA), average pore size, and the total pore volume of the AC-PPs using $ZnCl_2$ as activating agent with the corresponding B and N precursors $B_2O_3$ and urea. The reference material is AC-Peat.

| Sample | SSA [$m^2 \cdot g^{-1}$] | Average pore size [nm] | Total pore volume [$cm^3 \cdot g^{-1}$] |
|---|---|---|---|
| AC-Peat | 658.7 | 1.38 | 0.45 |
| AC-PPs | 825.3 | 1.00 | 0.41 |
| AC-PPs-B-N | 793.5 | 0.95 | 0.38 |
| AC-PPs-N | 770.0 | 1.00 | 0.38 |
| AC-PPs-B | 627.2 | 1.11 | 0.35 |

Table S6: EC in dependence of the pressure of the AC-PPs applying $ZnCl_2$ as activating agent with the corresponding B and N precursors $B_2O_3$ and urea. The reference material is AC-Peat.

| Sample | Pressure [kPa] | EC [$S \cdot m^{-1}$] |
|---|---|---|
| AC-Peat | i) 23.8<br>ii) 272.2<br>iii) 644.7<br>iv) 1265.6 | i) 8.9±2.0<br>ii) 22.7±4.4<br>iii) 37.0±3.4<br>iv) 58.3±4.8 |
| AC-PPs | i) 23.8<br>ii) 272.2<br>iii) 644.7<br>iv) 1265.6 | i) 11.0±1.6<br>ii) 12.8±1.2<br>iii) 13.6±2.0<br>iv) 25.5±0.8 |
| AC-PPs-B-N | i) 23.8<br>ii) 272.2<br>iii) 644.7<br>iv) 1265.6 | i) 9.2±2.1<br>ii) 11.7±1.0<br>iii) 20.8±1.4<br>iv) 34.0±2.5 |
| AC-PPs-N | i) 23.8<br>ii) 272.2<br>iii) 644.7<br>iv) 1265.6 | i) 2.6±0.3<br>ii) 10.4±0.7<br>iii) 18.1±0.8<br>iv) 26.1±1.5 |
| AC-PPs-B | i) 23.8<br>ii) 272.2<br>iii) 644.7<br>iv) 1265.6 | i) 2.0±0.07<br>ii) 20.8±0.7<br>iii) 33.7±2.9<br>iv) 50.3±4.4 |

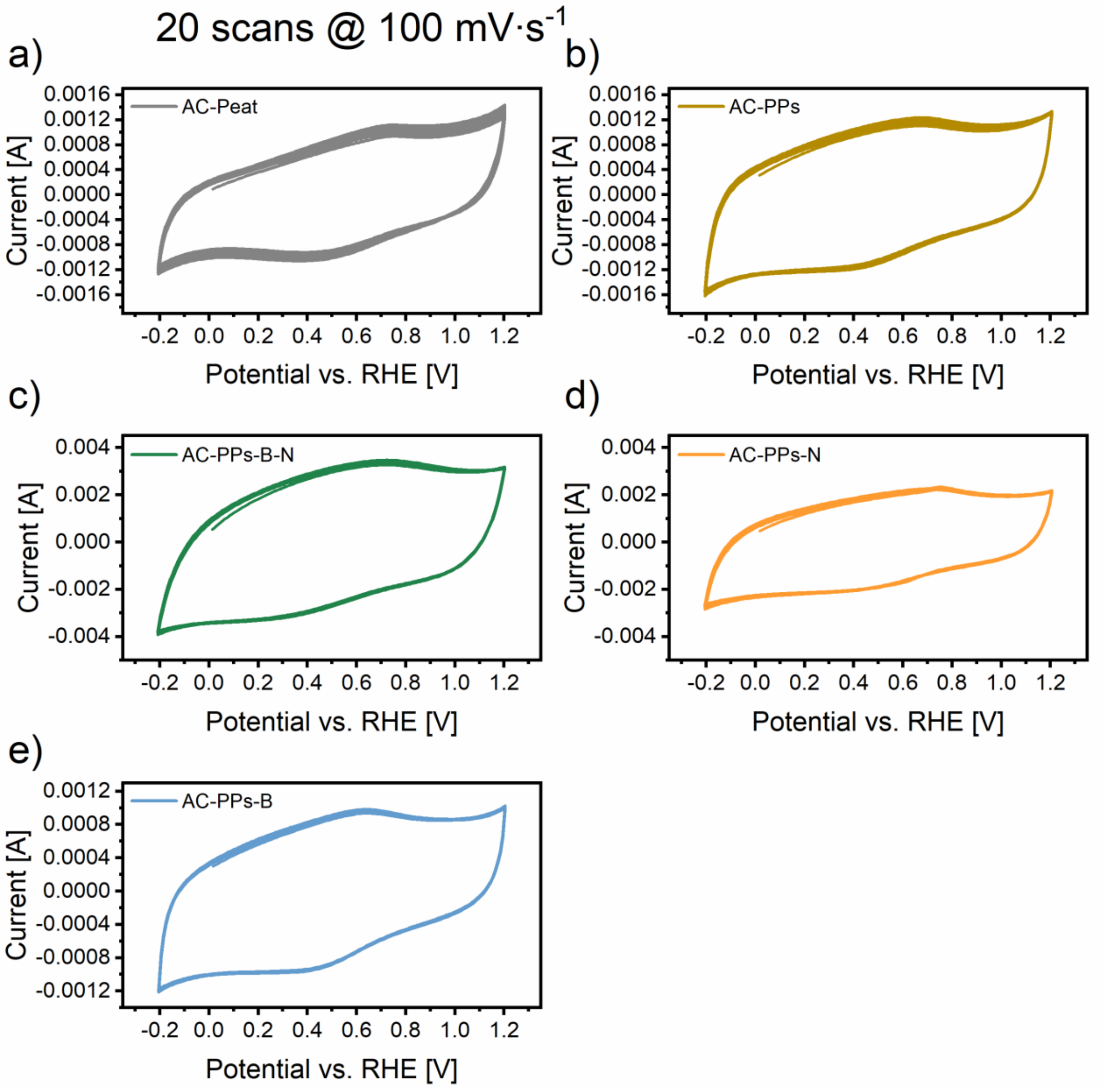


Figure S1: CV curves of the PPs-derived AC electrodes with the corresponding B and N precursors $B_2O_3$ and urea (b)-(e) compared with the reference material AC-Peat (a) after 20 scans at a scan rate of 100 mV·s$^{-1}$ in 1 M $H_2SO_4$.

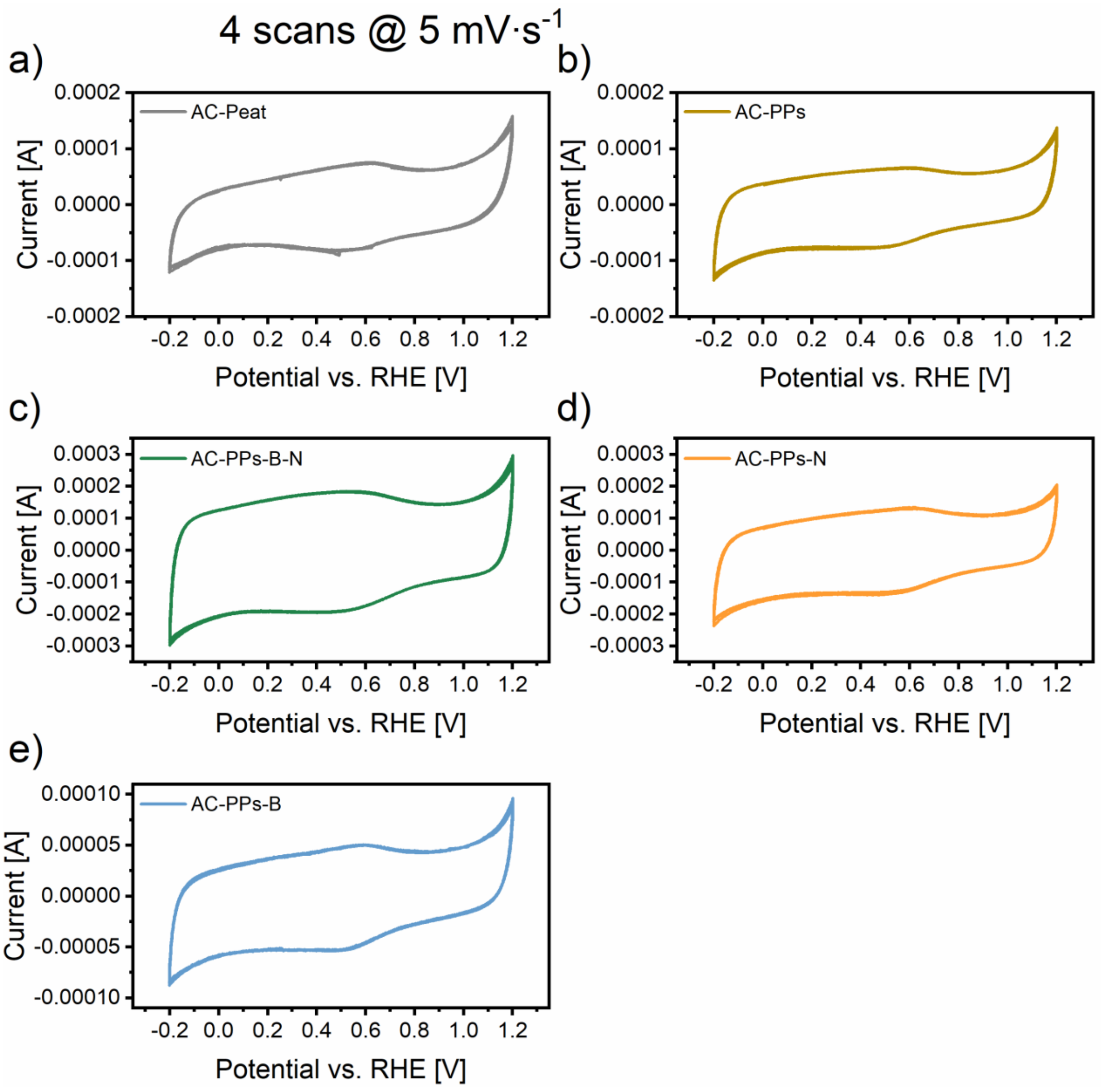


Figure S2: CV curves of the PPs-based AC electrodes with the corresponding B and N precursors $B_2O_3$ and urea (b)-(e) in comparison with the reference material AC-Peat (a) after 4 scans at a scan rate of 5 mV·s$^{-1}$ in 1 M $H_2SO_4$.

Table S7: Specific capacitances of the AC electrodes derived from PPs applying $ZnCl_2$ as activating agent with the corresponding B and N precursors $B_2O_3$ and urea at a scan rate of 100 mV·s$^{-1}$ and 5 mV·s$^{-1}$ in 1 M $H_2SO_4$. The reference material is AC-Peat.

| Sample | Specific capacitance at 100 mV·s$^{-1}$ [F·g$^{-1}$] | Specific capacitance at 5 mV·s$^{-1}$ [F·g$^{-1}$] |
|---|---|---|
| AC-Peat | 37.7±2.5 | 54.3±11.0 |
| AC-PPs | 27.7±0.7 | 39.5±3.3 |
| AC-PPs-B-N | 51.7±10.3 | 71.9±9.7 |
| AC-PPs-N | 32.8±5.9 | 48.1±3.5 |
| AC-PPs-B | 23.6±1.8 | 43.3±17.8 |

Table S8: Comparison of PPs-derived carbon electrodes.

| Electrode | Activation | SSA $[m^2 \cdot g^{-1}]$ | Specific capacitance $[F \cdot g^{-1}]$ | Electrolyte | Reference |
|---|---|---|---|---|---|
| (a) Pot<br>(b) Cu-Pc<br>(c) Cu-Pc/Pot | KOH@room temperature | n/a | (a) 61<br>(b) 163<br>(c) 237 | 1 M $H_2SO_4$ | [1] |
| (a) PAC<br>(b) S/PAC<br>(c) S, P/PAC | KOH@750 °C | (a) 1493.3<br>(b) 1645.8<br>(c) 1911.5 | (a) 98<br>(b) 211<br>(c) 323 | 1 M $Na_2SO_4$ | [2] |
| (a) $Cpp_6$<br>(b) $Cpp_7$<br>(c) $Cpp_8$ | (a) $ZnCl_2$/LiCl@600 °C<br>(b) $ZnCl_2$/LiCl@700 °C<br>(c) $ZnCl_2$/LiCl@850 °C | (a) 103.2<br>(b) 159.5<br>(c) 314.4 | (a) 283<br>(b) 343<br>(c) 603 | 0.5 M $H_2SO_4$ | [3] |
| (a) ST-0.1<br>(b) ST-0.3<br>(c) ST-0.5 | (a) 0.1 M $ZnCl_2$@600 °C in $N_2$ + 850 °C in $CO_2$<br>(b) 0.3 M $ZnCl_2$@600 °C in $N_2$ + 850 °C in $CO_2$<br>(c) 0.5 M $ZnCl_2$@600 °C in $N_2$ + 850 °C in $CO_2$ | n/a | (a) 83<br>(b) 196<br>(c) 147 | 1 M $H_2SO_4$ | [4] |

## References


[1] R. J. Wesley, A. Durairaj, S. Ramanathan, A. Obadiah, R. Justinabraham, X. Lv, S. Vasanthkumar, *Diamond Relat. Mater.* **2021**, *115*.

[2] D. Khalafallah, X. Quan, C. Ouyang, M. Zhi, Z. Hong, *Renewable Energy* **2021**, *170*, 60–71.

[3] N. Q. Abro, N. Memon, B. A. Samejo, M. R. Halepoto, A. A. Hakro, *Biomass Convers. Biorefin.* **2022**.

[4] D. Pertiwi, N. Yanti, R. Taslim, *J. Phys.: Conf. Ser.* **2022**, *2193*, 12019.
